# A trans locus causes a ribosomopathy in hypertrophic hearts that affects mRNA translation in a protein length-dependent fashion

**DOI:** 10.1186/s13059-021-02397-w

**Published:** 2021-06-28

**Authors:** Franziska Witte, Jorge Ruiz-Orera, Camilla Ciolli Mattioli, Susanne Blachut, Eleonora Adami, Jana Felicitas Schulz, Valentin Schneider-Lunitz, Oliver Hummel, Giannino Patone, Michael Benedikt Mücke, Jan Šilhavý, Matthias Heinig, Leonardo Bottolo, Daniel Sanchis, Martin Vingron, Marina Chekulaeva, Michal Pravenec, Norbert Hubner, Sebastiaan van Heesch

**Affiliations:** 1 Cardiovascular and Metabolic Sciences, Max Delbrück Center for Molecular Medicine in the Helmholtz Association (MDC), 13125 Berlin, Germany; 2 Present Address: NUVISAN ICB GmbH, Lead Discovery-Structrual Biology, 13353 Berlin, Germany; 3 Berlin Institute for Medical Systems Biology (BIMSB), Max Delbrück Center for Molecular Medicine in the Helmholtz Association (MDC), 10115 Berlin, Germany; 4 Present Address: Department of Biological Regulation, Weizmann Institute of Science, 7610001 Rehovot, Israel; 5 Present Address: Program in Cardiovascular and Metabolic Disorders, Duke-National University of Singapore, Singapore, 169857 Singapore; 6 DZHK (German Centre for Cardiovascular Research), Partner Site Berlin, 13347 Berlin, Germany; 7 Charité–Universitätsmedizin, 10117 Berlin, Germany; 8 Institute of Physiology of the Czech Academy of Sciences, 4, 142 20 Praha, Czech Republic; 9 Institute of Computational Biology (ICB), HMGU, Ingolstaedter Landstr. 1, 85764 Neuherberg, Munich, Germany; 10 Department of Informatics, Technische Universitaet Muenchen (TUM), Boltzmannstr. 3, 85748 Garching, Munich, Germany; 11 Department of Medical Genetics, University of Cambridge, Cambridge, CB2 0QQ UK; 12 The Alan Turing Institute, London, NW1 2DB UK; 13 MRC Biostatistics Unit, University of Cambridge, Cambridge, CB2 0SR UK; 14 Institut de Recerca Biomedica de Lleida (IRBLLEIDA), Universitat de Lleida, Edifici Biomedicina-I. Av. Rovira Roure, 80, 25198 Lleida, Spain; 15 Department of Computational Molecular Biology, Max Planck Institute for Molecular Genetics, 14195 Berlin, Germany; 16 Present Address: The Princess Máxima Center for Pediatric Oncology, Utrecht, the Netherlands

**Keywords:** Genetic variation, *trans* QTL mapping, Translational efficiency, Ribosome profiling, Cardiac hypertrophy, Ribosome biogenesis, Ribosomopathy, Complex disease, Spontaneously hypertensive rats (SHR), HXB/BXH rat recombinant inbred panel

## Abstract

**Background:**

Little is known about the impact of trans-acting genetic variation on the rates with which proteins are synthesized by ribosomes. Here, we investigate the influence of such distant genetic loci on the efficiency of mRNA translation and define their contribution to the development of complex disease phenotypes within a panel of rat recombinant inbred lines.

**Results:**

We identify several tissue-specific master regulatory hotspots that each control the translation rates of multiple proteins. One of these loci is restricted to hypertrophic hearts, where it drives a translatome-wide and protein length-dependent change in translational efficiency, altering the stoichiometric translation rates of sarcomere proteins. Mechanistic dissection of this locus across multiple congenic lines points to a translation machinery defect, characterized by marked differences in polysome profiles and misregulation of the small nucleolar RNA SNORA48. Strikingly, from yeast to humans, we observe reproducible protein length-dependent shifts in translational efficiency as a conserved hallmark of translation machinery mutants, including those that cause ribosomopathies. Depending on the factor mutated, a pre-existing negative correlation between protein length and translation rates could either be enhanced or reduced, which we propose to result from mRNA-specific imbalances in canonical translation initiation and reinitiation rates.

**Conclusions:**

We show that distant genetic control of mRNA translation is abundant in mammalian tissues, exemplified by a single genomic locus that triggers a translation-driven molecular mechanism. Our work illustrates the complexity through which genetic variation can drive phenotypic variability between individuals and thereby contribute to complex disease.

**Supplementary Information:**

The online version contains supplementary material available at 10.1186/s13059-021-02397-w.

## Background

Gene expression regulation is a multilayered process and variation at any level can influence susceptibility to disease [1, 2]. Heritable, naturally occurring genetic variation can induce gene expression changes through epigenetic [3–5], transcriptional [6–8], and post-transcriptional [9–13] mechanisms. However, the extent to which *trans*-acting factors influence mRNA translation and thereby contribute to phenotypic diversity between individuals, and possibly complex disease, is not known. In this study, we use the rat HXB/BXH recombinant inbred (RI) panel to identify distant genetic effects on mRNA translation in a complex disease-relevant setting. The HXB/BXH panel is a powerful and well-characterized model system for rat genetics that was established in 1989 [14] and consists of 30 RI lines, derived from crossing normotensive Brown Norway-*luxate* (BN-*Lx*) and spontaneously hypertensive rats (SHR/Ola; hereafter SHR) (*reviewed in* [15]). Each of these 30 RI lines possesses a homozygous mixture of the ± 3.6 million genetic positions that discriminate both parental lines [16, 17]. Within the HXB/BXH panel, these genetic variants can be associated with physiological and molecular phenotypes to uncover disease-relevant genotype-phenotype relationships [18–21]. Importantly, for each of the two parental genotypes (BN-*Lx* and SHR), any genetic locus is on average replicated by 15 out of 30 RI lines, providing sufficient power to detect not only local (*cis*) but also distant, *trans*-acting QTLs.

Here we defined the influence of genetic variation on the efficiency of mRNA translation (translational efficiency, or TE) by applying ribosome profiling (or Ribo-seq [22]) and RNA-seq to liver and left ventricular heart tissue of each of the 30 RI lines—two tissues directly related to the cardiovascular and metabolic traits present in SHR. Focusing specifically on distant translational efficiency QTLs (teQTLs), we discovered a prominent set of *trans-*acting “hotspots” that each controlled the translation of up to dozens of genes in the rat heart. Amongst these potential translational master regulators, we found a single distant teQTL on rat chromosome 3 that influenced TE in a translatome-wide and protein length-dependent fashion. In-depth investigation of this locus, which overlapped a highly replicated locus for left ventricular mass [20, 23, 24], revealed a defect in ribosome biogenesis that appears to induce polysome half-mer formation, the accumulation of higher-order polysomes on relatively short coding sequences, and misregulation of the most highly abundant small nucleolar RNA *SNORA48*. The ribosome deficiency induced by this genetic locus was specific to SHR hearts, where it reinforced a protein length-dependent imbalance in protein synthesis rates that existed at baseline [25–29], but was amplified in hypertrophic hearts. We continued to show that length-specific shifts in TE are a common and conserved hallmark of translation machinery defects, including the ones that commonly cause human ribosomopathies. We propose that mutations in translational machinery factors differ in their impact on translation initiation and closed-loop translation reinitiation, which either results in a positive or negative amplification of the at baseline negative correlation between protein-coding sequence length and the efficiency of mRNA translation.

With our work, we show how translation in mammalian tissues can be under extensive distant genetic control by a limited number of master regulatory loci. We highlight a single genetic locus that induces a complex, translation-driven molecular mechanism in rat hearts that contributes to phenotypic diversity and underlies a complex cardiac trait.

## Results

### Identification of translational efficiency QTLs in the HXB/BXH panel

**Fig. 1:**
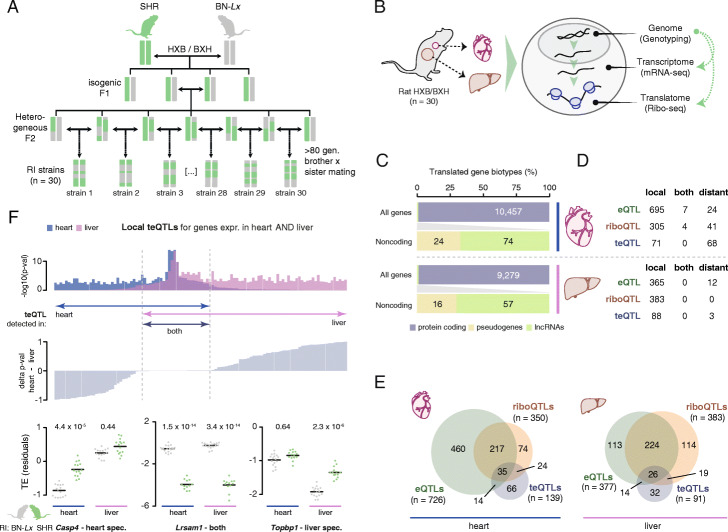
Identification of translational efficiency QTLs in the HXB/BXH panel. **A** Schematic overview of the establishment of the HXB/BXH recombinant inbred panel. Colored bars represent SHR and BN-*Lx* alleles. **B** Schematic overview of the experimental procedures carried out for each of the 30 HXB/BXH lines. **C** Bar plot illustrating absolute and relative ORF identifications, separated by coding and noncoding gene biotypes, for heart and liver. Translated pseudogenes were excluded from downstream analyses. See also Additional file 2: Table S1. **D** Table with gene-centric QTL mapping results for heart and liver, separated by genes for which mRNA expression QTLs (eQTLs), ribosome occupancy QTLs (riboQTLs), and/or translational efficiency QTLs (teQTLs) are identified. Local QTLs indicate associations that map to the same genomic locus as the tested gene (see “Methods”). Distant QTLs refer to associations with genes on chromosomes other than that of the QTL. See also Additional file 3: Table S2. **E** Venn diagrams displaying a gene-centric overlap of eQTLs, riboQTLs, and teQTLs in heart and liver, highlighting QTLs shared with, or specific to, a single trait. **F** Bar plot with a tissue comparison of detected local translational efficiency QTLs (teQTLs) considering only genes expressed and translated in both tissues. Genes are ordered by the delta p value in heart vs. liver tissue (middle panel). Three examples of genes expressed in heart and liver tissue are given, displaying a local teQTL in either one, or both, of these tissues. Cross bars indicate mean values. See also Additional file 1: Figures S1-S2 and Additional files **2**, 3, 4: Tables S1, S2, S3

To be able to associate genetic variation with translational efficiencies, we further refined a previously constructed [3, 30] genotype map of the HXB/BXH RI panel (see “Methods” and Fig. 1A). The obtained genotypes were associated with the mRNA expression and translation levels of 10,531 cardiac and 9336 liver genes (77% overlap), which were obtained using deep Ribo-seq and RNA-seq data across each of the 30 RI lines (Fig. 1B, C, Additional file 1: Figure S1A-H and Additional file 2: Table S1). We identified and categorized three types of QTLs per tissue: mRNA expression QTLs (eQTLs; mRNA-seq levels), ribosomal occupancy QTLs (riboQTLs; Ribo-seq levels), and translational efficiency QTLs (teQTLs; Ribo-seq levels corrected for mRNA-seq levels) (Fig. 1D, E, Additional file 1: Figure S1I-K and Additional file 3: Table S2). In line with previous work [9–11, 31], we found that most local QTLs had a clear transcriptional basis (i.e., as eQTLs) whose effect size and directionality was, with minor variations, concordantly visible in the Ribo-seq data (Additional file 1: Figure S2A + B). However, *cis* effects specifically significant to either mRNA expression or ribosomal occupancy were also observed (Fig. 1E and Additional file 1: Figure S2A + B). This specificity concordantly resulted in a set of genes with local teQTLs (n_heart_ = 71 and n_liver_ = 88), with expression changes induced during translational regulation in a manner independent of mRNA expression levels (Additional file 1: Figure S2A + C). These teQTLs showed limited recurrence between heart and liver, despite most genes with teQTLs being expressed in both tissues (see “Methods,” Fig. 1F, Additional file 1: Figure S2B and Additional file 3: Table S2). While this is possibly explained by liver being a frequent outlier in cross-tissue eQTL comparisons [32], these findings suggest that many *cis*-acting teQTLs are mediated in a tissue-specific manner.

It should be stressed that despite some effects being categorized as tissue- (e.g., heart or liver) or trait- (e.g., eQTL or riboQTL) specific based on our significance cutoffs, the non-significant counterpart within these comparisons frequently showed similar effect size directionality. For instance, 95% of local trait-specific QTLs (e.g., eQTL vs. riboQTL) and 82% of local tissue-specific QTLs (heart vs. liver teQTLs) shared the same effect directionality in the non-significant category, albeit with strongly reduced effect sizes (Additional file 1: Figure S2B). Such QTLs may have gone undetected because of reduced power within the HXB/BXH RI panel to detect traits with low effect sizes or low heritabilities (see “Methods,” Fig. 1F and Additional file 1: Figure S2A + B + D). Thus, although QTLs specific to tissues or traits could clearly be detected, our analyses did show that QTLs with low effect sizes can be missed. These could be considered false-negative QTLs, contributing to incomplete inferences of trait or tissue specificity.

### Local teQTLs are mechanistically independent of upstream ORFs

Upstream ORFs (uORFs) are major *cis* regulatory elements of translation located in 5′ leader sequences of protein-coding mRNAs [33], and genetic variants interfering with these elements can affect the efficiency of mRNA translation [10]. Out of over a thousand newly detected uORFs per tissue (Additional file 1: Figure S1H and Additional file 4: Table S3), we detected 27 (heart) and 13 (liver) uORFs whose translation rates associated with genetic variants in *cis* (“uORF-QTLs;” Additional file 4: Table S3). However, none of these variants disrupted the uORF’s start or stop codon, and only a single uORF-QTL mapped to a gene with a primary ORF teQTL. For this gene, *Rte1*, both QTLs showed the same effect directionality, indicating that increased translation of the uORF had no negative impact on the primary ORF TE (Additional file 1: Figure S2E). In general, uORF and primary ORF translation rates showed a very limited quantitative dependency (as observed in [31, 34–36]) (Additional file 4: Table S3, Additional file 1: Figure S2F + G) and we found no enrichment of uORFs in genes with local teQTLs (p_heart_ = 0.70 and p_liver_ = 0.79). In addition, we found no genetic variants in genes with local teQTLs that interfered with local translation initiation context or Kozak sequence, although effects may have been too subtle to detect. Similarly, we could not determine the possible outcome of genetic variants in other functional elements that serve to fine-tune mRNA translation, such as RNA folding structures, methylation sites, or RNA-binding protein motifs [37, 38]. Combined, our observations imply that uORFs are unlikely to be main drivers of local teQTLs within the HXB/BXH panel.

### Distant teQTL “hotspots” are master regulators of cardiac translation

**Fig. 2:**
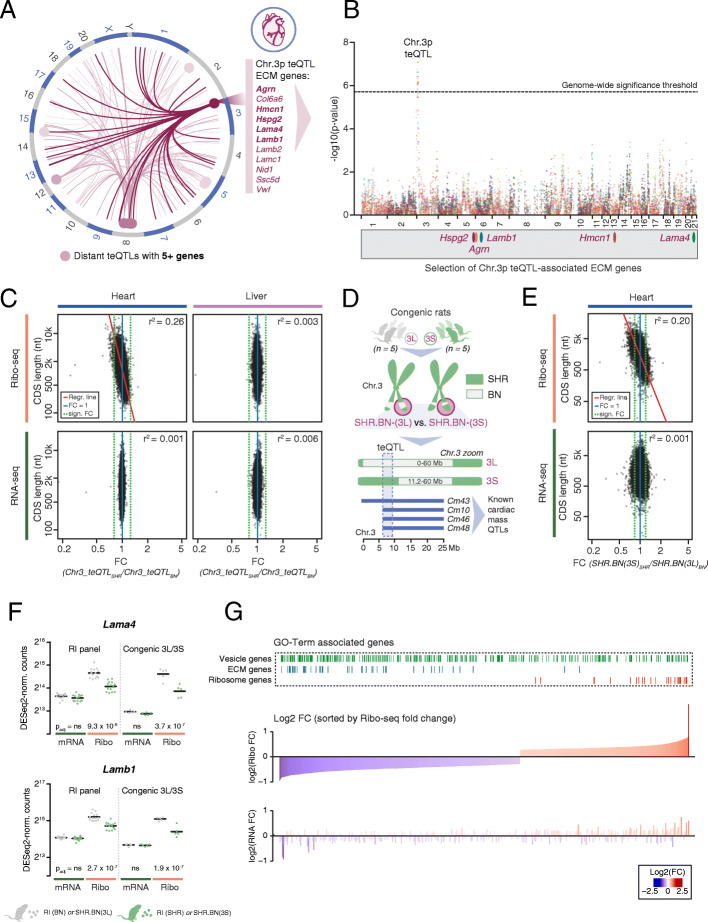
The chromosome 3p teQTL regulates cardiac translation in a protein length-dependent manner. **A** Circos plot highlighting all distant teQTLs and gene-locus associations detected in the rat heart that associate with the TE of at least 5 genes. The Chr. 3p teQTL is highlighted in dark pink and of the 25 associated genes, only the names of the 11 extracellular matrix (ECM) genes are given. **B** Overlay of Manhattan plots displaying genome-wide significance values for a genetic association with TE on Chr. 3p. A selection of 5 associated genes whose protein products function in the extracellular matrix are shown. **C** Scatter plots and square correlation coefficients (r^2^) based on standardized major axis (SMA) values between coding sequence (CDS) length and the fold change (FC) in gene expression, as measured by Ribo-seq (top) or mRNA-seq (bottom), for heart (left) and liver tissue (right). To define the expression FC, all 30 RI lines are separated by local genotype (BN or SHR) at the Chr. 3p teQTL. For heart Ribo-seq data, the correlation is significant (p value < 2.2 × 10^−16^; test of correlation coefficient against zero) and the linear model based on fitted SMA method is displayed as a red line. **D** Schematic overview of the congenic rat lines with isolated teQTL and cardiac mass QTL locus. The SHR.BN-(3L) line carries a local BN genotype, whereas the SHR.BN-(3S) line retains the SHR genotype at the teQTL. Inserted BN segments are visualized in grey, SHR alleles in green. **E** Scatter plots and square correlation coefficients (r^2^) based on standardized major axis (SMA) values between coding sequence (CDS) length and the fold change (FC) in gene expression, as measured by Ribo-seq (top) or mRNA-seq (bottom) in congenic rat hearts. The FC in translation is derived from a comparison between 5 replicates of SHR.BN-(3L) and SHR.BN-(3S) rats and reproduces the global length effect observed for the Chr. 3p teQTL identified in the HXB/BXH RI panel. For heart Ribo-seq data, the correlation is significant (p value < 2.2 × 10^−16^; test of correlation coefficient against zero) and the linear model based on fitted SMA method is displayed as a red line. **F** Dot plots with indications of mean expression for 2 laminin subunits (extracellular matrix glycoproteins), illustrating the reproducibility of the translational efficiency phenotype between the HXB/BXH RI panel and the congenic rat lines. Cross bars indicate mean values. **G** Bar plot with all differentially translated genes in a comparison of both congenic rat lines, ordered by Ribo-seq FC in expression. Genes associated with selected significant GO terms are highlighted on top. See also Additional file 1: Figure S3 and Additional file 5: Table S4

Distant QTLs are an important source of variation in mRNA expression levels, through which they contribute to complex disease [32, 39]. Although the impact of *trans*-acting QTLs on mRNA translation in a complex disease setting has remained unexplored, the HXB/BXH panel provides enough power to detect such QTLs [7, 18, 21, 40]. Because we found distant teQTLs to be more frequent in heart than in liver (Fig. 1D and Additional file 1: Figure S2B), we decided to focus downstream analyses solely on heart tissue. To increase the power to detect genes with shared modes of regulation by a single QTL “hotspot,” we applied a hierarchical regression model in a Bayesian framework using a stochastic search algorithm (HESS [41, 42]) (see “Methods”). This approach yielded a higher total of 243 genes whose TE is regulated by a distant teQTL (Additional file 1: Figure S3A and Additional file 5: Table S4). Of all distant teQTLs, we classified nine loci as distant cardiac master regulators, as they influenced the TE of at least 5 (but up to 25) genes distributed over different chromosomes (Fig. 2A, Additional file 1: Figure S3B and Additional file 5: Table S4).

A single 2.9 Mb large teQTL hotspot on rat chromosome 3p (Chr. 3 6.3–9.2 Mb; equivalent to human Chr. 9q34) drew our attention for being associated with the TE of 25 genes (Fig. 2A, Additional file 1: Figure S3B, Additional file 3: Table S2 and Additional file 5: Table S4). This locus furthermore matched a highly replicated QTL for cardiac mass [23, 24], for which a loss-of-function insertion in endonuclease G (*Endog*) was previously identified as a driver of cardiomyocyte hypertrophy and increased left ventricular weight [20]. Among all genes associated with this master regulatory teQTL, we found strong enrichment for extracellular matrix (ECM) proteins (11 out of 25; GO:0031012; p_adj_ = 3.32 × 10^−10^) (Fig. 2A, B), consistent with recent observations of strong translational control of fibrotic processes in human hearts [31, 43].

### The chromosome 3p teQTL regulates cardiac translation in a protein length-dependent manner

The strong translational impact on ECM genes led us to hypothesize that the differential translation could be related to a global switch in translational control related to the generally high coding sequence (CDS) length of ECM proteins. Indeed, we observed a moderate, though highly significant correlation between CDS length and fold change (FC) in translation (r^2^ = 0.26; p < 2.2 × 10^−16^), which produces a downregulatory effect for genes with long CDSs and, vice versa, an upregulatory effect for genes with short CDSs (Fig. 2C). This association with CDS length was specific to heart tissue, absent in RNA-seq data, and no other genetic locus outside of the Chr. 3p teQTL showed a similar effect.

To replicate this translatome-wide phenotype, we performed ribosome profiling on two congenic rat lines with two small, but differently sized, BN segments inserted into the short arm of Chr. 3 on an otherwise fully SHR background [20] (see “Methods” and Fig. 2D). The first congenic line possessed a long BN segment that replaced the teQTL completely (SHR.BN-(3L)), whereas the second line contained a smaller BN segment positioned adjacent to the teQTL (SHR.BN-(3S)), hence leaving the teQTL intact. Comparing the cardiac translatomes of both congenic lines, we fully recapitulated the protein length-dependent difference in translation observed in the HXB/BXH RI panel (r^2^ = 0.20; p < 2.2 × 10^−16^; Fig. 2E, F). A subsequent GO enrichment analysis on differentially translated genes concordantly yielded terms matching the downregulation of very large proteins (GO: extracellular region; p_adj_ = 6.33 × 10^-13^) or the upregulation of very small proteins (GO: cytosolic ribosome; p_adj_ = 1.22 × 10^-13^) (Fig. 2G). Of note, the observed TE fold changes specifically correlated with CDS length (r^2^ = 0.20), to a lesser extent with total transcript length (r^2^ = 0.162) but not with 5′ UTR (r^2^ = 0.004) or 3′ UTR length (r^2^ = 0.013) (Additional file 1: Figure S3C).

### The chromosome 3p teQTL induces changes in mono- and polysome occupancy that impact stoichiometric sarcomere translation

**Fig. 3:**
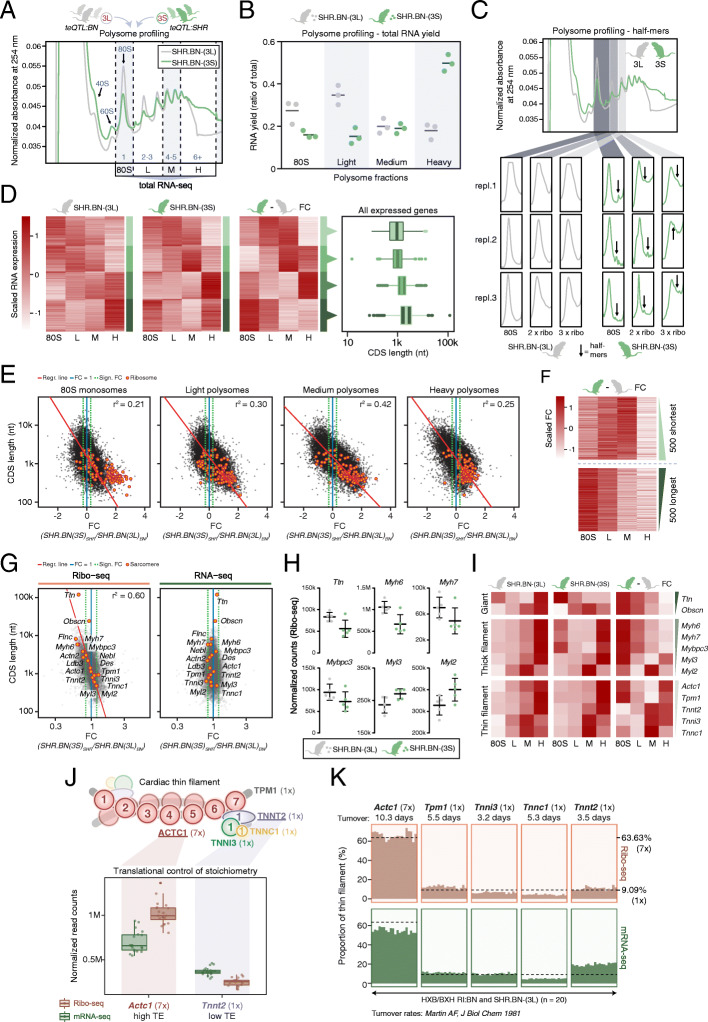
The chromosome 3p teQTL induces polysome half-mer formation. **A** Schematic overview of the polysome fractionation and RNA-seq approach. One representative polysome profile per congenic rat line is given. L, M, and H fractions indicate light, medium, and heavy polysomes, respectively. **B** Congenic line comparison for differences in the number of associated ribosomes per mRNA, as measured by the distribution of RNA yield across the fractions. Quantified polysome profile area under curves (AUCs) can be found in Additional file 1: Figure S4A. Bars indicate mean values. **C** Zoomed-in view of multiple polysomal peaks across replicates for both congenic lines, with arrows indicating possible half-mers. **D** Heatmap with scaled RNA-seq expression levels of all 12,471 quantified genes (mean RNA FPKM ≥ 1 across replicates, for both lines). Genes are clustered into 4 groups by k-means clustering and sorted by CDS length within each cluster. The same gene order obtained through clustering of the fold change (SHR.BN-(3S) vs SHR.BN-(3L)) comparison (3rd heatmap) was used for the individual heatmaps of SHR.BN-(3L) vs SHR.BN-(3S) (1st and 2nd heatmap). For all clusters, box plots with the CDS length distribution are shown on the right. **E** Scatter plots and square correlation coefficients (r^2^) based on standardized major axis (SMA) values between coding sequence (CDS) length and the fold change in gene expression (FC (SHR.BN-(3S) vs SHR.BN-(3L)), as measured by RNA-seq of the four isolated fractions. The correlations are significant (p value < 2.2 × 10^−16^; test of correlation coefficient against zero) and the linear model based on fitted SMA method are displayed as red lines. Ribosomal protein genes (with small CDSs) are depicted by orange dots. **F** Heatmaps with the scaled FC of the ribosomal configuration of the top 500 shortest and longest CDS genes. **G** Scatterplots showing CDS length versus fold change (FC (SHR.BN-(3S) vs SHR.BN-(3L)) for Ribo-seq and RNA-seq data, highlighting a representative selection of core- and accessory sarcomere proteins. The square correlation coefficient (r^2^) based on standardized major axis (SMA) is calculated using expression values of this subset of genes only. **H** Dot plots with Ribo-seq expression values for *Ttn* and a selection of cardiac thick filament proteins. Genes are sorted by CDS length from top left to bottom right. Error bars indicate mean values with standard deviation (SD). None of the displayed expression changes are genome-wide significant. **I** Heatmaps with polysome profiling results for selected sarcomere proteins. Expression distributions for the individual animals, as well as the scaled fold changes between SHR.BN-(3S) and SHR.BN-(3L), are given. Within each group, genes are sorted by CDS length (top to bottom). **J** Schematic representation of the cardiac thin filament and its composition stoichiometry as obtained from [44]. Cardiac muscle alpha actin (*Actc1*) and cardiac troponin T (*Tnnt2*) are the genes most strongly translationally regulated to achieve desired protein levels. **K** Bar plots showing the relative contribution of each thin filament component as measured by Ribo-seq (top) and mRNA-seq (bottom) expression levels. DESeq2-normalized expression values are corrected for reported rat heart protein turnover rates [45] and represented as a percentage of the complete thin filament. Twenty healthy rats are shown (from left to right: 5× SHR.BN-(3L) congenic animals, followed by 15× HXB/BXH RI lines as separated by local BN genotype according to the Chr. 3p teQTL). Optimal production values for 7 or 1 subunit(s) are indicated by dashed lines. See also Additional file 1: Figure S4

To mechanistically dissect the translational phenotype linked to the Chr. 3p teQTL, we next performed polysome profiling on heart tissue of both congenic lines (Fig. 3A). Polysome profiles of SHR.BN-(3S) rats showed heavily altered differences in the numbers of ribosomes associated with mRNAs compared to SHR.BN-(3L) (Fig. 3A, B and Additional file 1: Figure S4A), additionally displaying small “shoulders” accompanying each mono- and poly-ribosome peak likely indicative of polysome half-mer formation (Fig. 3C) [46, 47]. Polysome half-mers are formed when the 43S preinitiation complex does not instantly join the large 60S ribosomal subunit to form a functional 80S monosome. This stalls translation initiation—the rate-limiting step of RNA translation and therefore a main determinant of TE [29, 48, 49]. Half-mers arise because of ribosome biogenesis defects, caused by the underproduction of 60S subunits [46] or impaired subunit joining [50, 51]. However, production levels of ribosomal RNA and protein components of both ribosomal subunits appeared balanced (Additional file 1: Figure S4B). SHR.BN-(3S) rats additionally showed increased accumulation of higher-order (heavy) polysomes, possibly indicative of a problem with translation termination or reflecting increased translation rates of mRNAs with short- or medium-size CDSs.

To evaluate these possibilities, we compared RNA-seq data from isolated fractions of monosomes (80S), light- (2–3 ribosomes), medium- (4–5 ribosomes), and heavy-weight polysomes (6+ ribosomes). This again revealed a clear relationship between ribosome occupancy and CDS length (Fig. 3D). This length dependency was identical to the one observed in the Ribo-seq data, validating the TE phenotype through an independent method (Fig. 3E). Whereas mRNAs with the longest CDSs showed a clear reduction in heavy polysome occupancy, accompanied by a relative enrichment in the monosomal fraction, mRNAs with the shortest CDSs showed increased steady-state translation in light- and medium polysomal configurations (Fig. 3F). As with all sequencing-based quantification experiments, measured differences are relative between fractions, as the RNA content of each sequenced library is normalized prior to comparison across fractions. This makes fraction-specific RNA-seq data suitable for comparing relative distributions and complexity of mRNAs across fractions, but less so for absolute quantitative comparisons between strains.

Among the genes most strongly affected by the length-dependent shift in ribosomal occupancy and TE were multiple core sarcomere proteins (Fig. 3G–I). These primarily included “giant” proteins *Ttn* and *Obscn*, as well as the larger protein constituents of the thick (*Myh6*, *Myh7*, and *Mybpc3*) and thin filament (*Actc1* and *Tpm1*), which all showed downregulated translation. In contrast, the much smaller components of the thick and thin filament, such as the myosin light chains (*Myl2* and *Myl3*) and cardiac troponins (*Tnnc1*, *Tnnt2*, and *Tnni3*), were all translationally upregulated. The large variability in sarcomere protein sizes correlated well with translational fold change (*r*^2^_sarcomere_ = 0.60; Fig. 3G), highlighting the impact of the Chr. 3p teQTL on sarcomere gene translation.

Of note, sarcomere homeostasis strongly depends on stoichiometric protein production and mRNA translation has been proposed, but not experimentally shown, to regulate this equilibrium [52, 53]. For the cardiac thin filament in particular, we indeed saw prominent translational control of protein production, exemplified by the translational up- and downregulation of *Actc1* (TE = 1.50) and *Tnnt2* (TE = 0.69), respectively, to achieve protein production levels in compliance with composition stoichiometry (Fig. 3J, K). In diseased hearts, the normally proportional filament translation rates are pushed into opposite directions because of differences in subunit CDS lengths (Fig. 3G–I). This makes it challenging to achieve composition stoichiometry in an energy-efficient manner [54, 55], as such imbalances need to be corrected post-translationally through the targeted degradation of excess subunits [56, 57].

### Imbalances between translation initiation and reinitiation reinforce a pre-existing length bias in TE

**Fig. 4:**
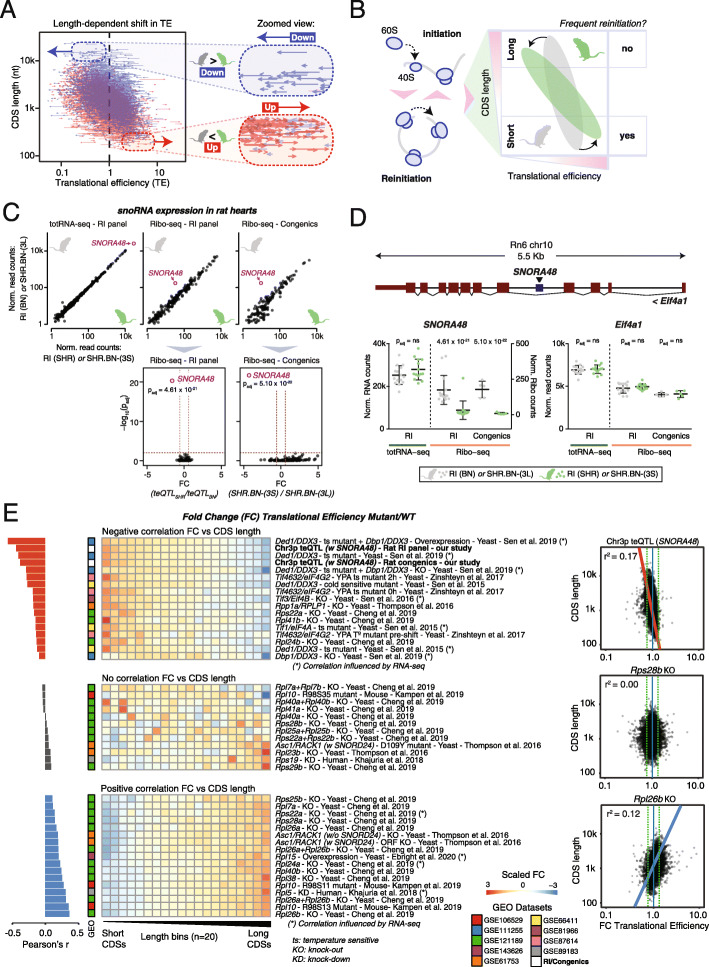
Imbalances between translation initiation and reinitiation reinforce a pre-existing length bias in TE. **A** Arrow-based scatter plot show the transitions in TE per gene, between SHR.BN-(3S) and SHR.BN-(3L) rats. The length of the arrow is representative of the absolute change in TE between both congenic lines, with the position of the arrow tail reflecting the SHR.BN-(3L) TE and the position of the arrowhead indicating the TE in SHR.BN-(3S) rats. Blue arrows indicate a decrease in TE in SHR.BN-(3S) rats, whereas red arrows indicate an increase in TE in SHR.BN-(3S). Two zoomed-in regions show arrow behavior in the top and bottom of the graph, respectively highlighting genes with very long and short CDSs. **B** Schematic of how ribosome biogenesis defects can lead to a change in translation initiation and reinitiation rates, driving a global shift in TE that correlates with CDS length. **C** Scatter plots showing expression levels of all cardiac-expressed snoRNAs as measured by totRNA-seq and Ribo-seq data, with *SNORA48* highlighted in pink. For both Ribo-seq datasets, *p* value volcano plots show the significance of the differential regulation of *SNORA48* (highlighted in pink). **D** Representation of the genomic location of *SNORA48*. This snoRNA is contained within intron 4 of its host gene *Eif4a1*. Dot plots with expression levels as measured by totRNA-seq and Ribo-seq for *SNORA48* and its host gene *Eif4a1*, in both the HXB/BXH RI panel and the congenic rat lines. Error bars indicate mean values with standard deviation (SD). See also Additional file 1: Figure S5. **E** Heatmap with CDS length versus fold change in TE (FC Mutant vs Wild Type) calculated from public Ribo-seq and RNA-seq data from various translational machinery mutants. Scaled fold changes are given. Within each group, genes are divided into 20 equally sized bins by increasing CDS length (left to right). Samples are sorted by Pearson’s correlation coefficient (r, top to bottom). Datasets are grouped as “negative correlation” or “positive correlation” depending on whether Pearson’s correlation coefficient value is lower than − 0.1 or higher than 0.1. The remaining datasets that showed no global shift in TE are grouped as “no correlation.” Scatter plots and Pearson’s square correlation coefficients (r^2^) between total CDS length and the FC in TE are displayed for three selected samples with one of the strongest negative Pearson’s r correlations (our model, chr3p teQTL), no correlation (*Rps28b* yeast knockout), and the strongest positive Pearson’s r correlation (*Rpl26b* yeast knockout)

Having established that the Chr. 3p teQTL causes a ribosomopathy that influences TE through changes in the number of translating ribosomes per mRNA and possibly through the formation of polysome half-mers, it remained unclear why the severity of this phenotype correlated with protein length. It is known that—even under normal conditions—the density of ribosomes along mRNAs is inversely correlated with CDS length and, as a consequence, longer proteins are generally less efficiently translated than shorter ones [25–28]. This length effect is directly coupled to the frequency of translation reinitiation, which decreases with increasing CDS length [27–29]. Indeed, in both SHR.BN-(3L) and SHR.BN-(3S) rat hearts, TE correlates negatively with CDS length (r^2^ = 0.12 and r^2^ = 0.21 respectively; Fig. 4A). Upon limited or hampered initial assembly of 80S monosomes, mRNAs would become increasingly dependent on effective ribosome recycling [28], for which both previously acquired ribosomal subunits remain instantly available. In agreement with this, the effect of CDS length on TE is significantly enhanced in SHR.BN-(3S) rats (Fisher r-to-z transformation Z = − 11; p < 2.2 × 10^−16^) (Fig. 4A). This effect is less detrimental for mRNAs with short CDSs, which more frequently reinitiate as one round of translation takes less time to complete, thereby reinforcing a pre-existing length dependency in translation (Fig. 4B). This also explains the increase in the number of ribosomes associated with short- and medium-sized CDS mRNAs in SHR.BN-(3S) relative to SHR.BN-(3L) rats (Fig. 3D, F).

According to the ribosome concentration hypothesis, changes in the absolute availability of functional ribosomes (e.g., as caused by ribosome biogenesis defects) can cause some mRNAs to respond differently to altered ribosome availability than others, in a manner dependent on mRNA-specific intrinsic translation initiation rates [58, 59]. Upon a reduction in the number of functional ribosomes, the TE of (classes of) mRNAs with poor initiation rates—e.g., transcription factors, mRNAs with highly structured 5′ UTRs or mRNAs with translated uORFs [58, 60]—has been proposed to be most strongly impacted. Although it is likely that the translational deficiency observed in hypertrophic hearts is accompanied by a reduction in overall ribosome availability, we found no enrichment for the previously proposed functional gene classes (e.g., transcription factors (Additional file 1: Figure S4C) or genes with uORFs (Additional file 1: Figure S4C) among the genes with the most strongly affected TE. Furthermore, 5′ UTR structure did not affect the observed phenotype (Additional file 1: Figure S4D). Therefore, we deem it unlikely that the aforementioned factors previously suggested to be the key determinants of mRNA-specific initiation rates are the main drivers of the response seen in the hypertrophic hearts.

The efficiency of translation and modes of translation initiation can also be impacted by proteotoxic stress at the endoplasmic reticulum (ER). Although ER stress and the unfolded protein response play a pivotal role in the pathophysiology of the heart [61, 62], we found no characteristic signatures of such a response: the polysome profiles did not resemble those typically observed upon ER stress (a strong shift from polysomal to predominantly monosomal translation [63, 64]) (Fig. 3A), translation elongation rates remained constant along the entire CDS [65] (Additional file 1: Figure S4E), protein levels of common ER stress markers (e.g., p-IRE1α:IRE1α ratios and XBP1s [66]) were unchanged (Additional file 1: Figure S4F and Additional file 1: Figure S6), and mRNA expression and translation levels of genes associated to ER stress (GO: 0034976) or apoptosis (GO: 0097190) were not affected (p values = 0.70 and 0.32, respectively).

Our results indicate that the observed mRNA-specific shift in translational efficiency results from an imbalance in translation initiation and reinitiation rates, which appears to primarily be determined by CDS length. Other factors that can each influence translational efficiency in an mRNA-specific manner, such as local regulatory elements, functional gene classes, or global stress responses, do not appear to be the main drivers of this effect.

### The highest expressed cardiac snoRNA *SNORA48* is misregulated in hypertrophic hearts

We next searched for possible misregulated ribosome biogenesis factors that could explain the observed phenotype, since mutations in these elements are known to induce global changes in polysome profiles similar to what we observe in the affected hearts [47, 67]. We find one such factor differentially regulated in the affected hearts: the H/ACA box small nucleolar RNA (snoRNA) *SNORA48* (also known as *ACA48*; Ensembl ID ENSRNOG00000060816). *SNORA48* is a conserved snoRNA predicted to guide the pseudouridylation (Ψ) of 28S ribosomal RNA (rRNA) during large ribosomal subunit biogenesis [68] (Additional file 1: Figure S5A). It was the most highly expressed snoRNA in rat hearts (Fig. 4C) and the only snoRNA that showed a genome-wide significant decrease in the Ribo-seq data, while overall production levels of the snoRNA and the host gene *Eif4a1* remained constant (Fig. 4D). SnoRNAs are a common bycatch in Ribo-seq datasets [69] and although difficult to determine, their presence may come from alternative non-ribosomal ribonucleoprotein complexes captured during the isolation of ribosome footprints [70], or reflect cytosolic ribosomal association induced by stress conditions [71].

To identify the cause of *SNORA48* misregulation, we investigated the involvement of the key Chr. 3p teQTL candidate gene *Endog*, whose loss-of-function (LoF) increases cardiac mass and impairs cardiac energy metabolism [20] (Additional file 1: Figure S5B). We profiled the cardiac translatomes of wild type and knockout *Endog* mice (obtained from [20]; 5 biological replicates each), as well as those of wild type and newly established transgenically rescued *Endog* SHR rats (Additional file 2: Table S1 and methods; 5 biological replicates each). These models showed no reduced levels of *SNORA48* in the Ribo-seq data and no clear length-dependent translational phenotype (Additional file 1: Figure S5C). This excludes *Endog* as a monogenic driver of the Chr. 3p teQTL and points to other mutated genes in the locus. Prime locus candidates with predicted damaging mutations include the DEAD box helicase *Ddx31* (yeast *DBP7*), whose deletion reduces 60S levels and induces half-mer formation in yeast [67, 72], the ribosomal RNA transcription termination factor *Ttf1* [73], or the methyltransferase *Spout1* (also known as *C9ORF114*), which codes for an essential pre-rRNA processing factor [67]. Although our data indicate that *Endog* does not act autonomously in the establishment of the Chr. 3p teQTL, complex genetic interactions with one or more of these dysfunctional ribosome biogenesis genes may be required for the translational phenotype to arise.

### Ribosomopathies induce length-specific shifts in TE that can move in opposite directions

A number of other studies, including work on human ribosomopathies such as Diamond-Blackfan anemia (DBA), have previously witnessed transcript-length-dependent changes in TE in the translatomes of various translation machinery mutants [58, 74–81]. Interestingly, some of these mutants resulted in the upregulation of short CDSs and downregulation of long CDSs (similar to the rat models studied here), whereas others showed opposite behavior, resulting in a reduced TE for short CDSs and increased TE for mRNAs with long CDSs. These contrasting observations led us to question whether there is a unifiable model that explains, and possibly predicts, the mutant-specific direction of this length effect. For this, we hypothesized that the mutated or dysfunctional component of the translation machinery should either more strongly impact the efficiency of translation initiation or reinitiation (e.g., by being required for the formation of closed loops). To test this hypothesis, we reanalyzed Ribo-seq and RNA-seq data of nine studies [75, 78–80, 82–86] that have investigated various translational machinery mutants, ranging from core ribosomal proteins to canonical eukaryotic initiation factors or accessory proteins. We compared each mutant with the respective control samples within each study and visualized the directionality and extent of the length effect alongside our rat model data. The resulting heatmap showed that many, but not all translation machinery mutants caused a significant, length-specific shift in translational efficiencies equally visible in yeast, human, mouse, and rat data (Fig. 4E). Remarkably, our rat models displayed among the most prominent negative length-specific TE shifts across all studied factors and species—even stronger than those observed for most knockout yeast models. It should be stressed that for a small subset of samples obtained from other studies, length-specific RNA-seq biases (in part) influenced TE calculations (Additional file 1: Figure S5D). Strikingly, other mutants that behaved similarly to our studied phenotype included the yeast canonical translation initiation factors and DEAD box RNA helicases Ded1 and Dbp1 (DDX3 family in mammals), Tif1 (eIF4A in mammals), and the non-helicase initiation factor Tif3 (eIF4B in mammals) and translational repressor Tif4632 (eIF4G2 in mammals), corroborating a likely initiation deficiency in our rat models. In contrast, factors previously found to be important for the translation of short CDSs because of their role in closed loop formation (e.g., Asc1/RACK1 [79]) and thus ribosome recycling, showed opposite translatome-wide CDS length-dependent shifts in TE. Similarly, a length effect in the same direction as the Asc1/RACK1 KO had been observed in microarray data from Tif4631/eIF4G1 KOs, which was proposed to be related to 5′ UTR structure [77]. Instead, our results lead us to propose a model that couples the directionality and extent of the change in TE to mRNA-specific difference initiation and reinitiation frequencies that primarily depend on CDS length.

In conclusion, we show that the loss of many translation machinery factors affects mRNA translational efficiency in a CDS length-dependent manner. We put forward a model where the process most strongly hampered by the mutant—either translation initiation, or reinitiation through ribosome recycling and the formation of closed loop mRNAs—defines the directionality of the length effect.

## Discussion

In this study, we used a QTL mapping strategy to define the influence of natural genetic variation on the efficiency of mRNA translation, with a focus on identifying distant, *trans*-acting QTLs that control multiple genes. Genetic influences on translation have previously been studied in yeast [11, 12] and a cohort of human lymphoblastoid cell lines (LCLs) [9, 10], albeit solely carried out in in vitro culture systems with limited focus on distant QTLs. These studies showed that within the investigated systems, the vast majority of (local) eQTLs were fed forward into variation in protein levels, with limited specific impact on translation ([9–12]; *reviewed in* [2, 87]). Although we similarly saw high concordance between local genetic influences on mRNA expression and translation, we did detect multiple teQTLs with specific and prominent effects on the mammalian tissue gene expression landscape. The most apparent and specific effects are orchestrated through a limited set of distant master regulatory loci—or teQTL “hotspots”—each controlling the TE of up to dozens of genes. This widespread distant translational control was particularly abundant in the heart and likely crucial for the adaptation to developing (patho)physiological conditions, though may have been dormant (and hence gone undetected) in unaffected tissue.

We mechanistically dissected a prominent distant teQTL on rat chromosome 3 that drove a translatome-wide and protein length-dependent change in TE. We showed that this teQTL induced a global shift in mono- and polysomal occupancy of mRNAs, accompanied by the formation of subtle though visible polysome half-mers. In diseased rat hearts, higher-order polysomes appeared to accumulate at mRNAs with small- or medium-sized CDSs to increase translational output, whereas mRNAs with longer CDSs showed opposite behavior and a strongly reduced TE. Even though all ribosomopathies originate from defects in ribosome biogenesis, they often lead to unique phenotypes with tissue-specific clinical manifestations [88]. This ribosomopathy is genetically induced in rats with a local SHR genotype in the Chr3 teQTL region, where its expressivity is specific to cardiac tissue and absent in liver. As another characteristic of this teQTL, we observed a striking reduction of *SNORA48* abundance in the Ribo-seq data. Although experimental evidence is as yet absent from current literature, the snoRNA *SNORA48* has been predicted to guide the modification of the large ribosomal subunit’s 28S rRNA backbone. Its highest abundance of all snoRNAs in the rat heart, and possibly in the hearts of other species, could indicate that its misregulation is detrimental for cardiac ribosome biogenesis.

Through reanalysis of public ribosome profiling datasets, we showed that several translation machinery mutants such as knockouts of the yeast translation initiation factors Ded1 (DDX3-like), Dbp1 (DDX3-like), Tif1 (eIF4A), and Tif3 (eIF4B) produced translational shifts similar to our rat models. Surprisingly, *SNORA48* is located in the intron of *Eif4a1*, the mammalian ortholog of *Tif1*, but the gene is intron-less in yeast. This raises the possibility of an evolutionary gained functional codependency between this initiation factor and *SNORA48* in mammalian translational regulation. Mutation of all of the abovementioned translation initiation factors resulted in the upregulated translation of short CDSs, whereas long CDSs were downregulated. In contrast, several other translation machinery mutants displayed the exact opposite profile. For instance, the snoRNA *SNORD24* (also known as *SNR24* or *U24*), whose KO in yeast also induced a polysome half-mer phenotype [47, 89], reduced the TE of short CDSs when deleted as part of its host gene *Asc1/RACK1*—a known closed loop factor [79].

In previous studies, the translational shifts upon mutation of these factors were generally explained as a specific preference or clear requirement of the investigated factor (or the specialized ribosome that has this factor incorporated) for a subset of mRNAs (e.g., short mRNAs [79], mRNAs of genes involved in specific pathways [74], long mRNAs with structured UTRs [78], or mRNAs with specific Internal Ribosome Entry Site (IRES) elements [90]). These effects were subsequently proposed to be caused by a reduced ability of the mutant to translate mRNAs with highly structured 5′ or 3′ UTRs, ORF length-dependent changes in the efficiency of closed-loop formation, or differences in overall ribosome concentrations [58, 74–81]. Our study provides evidence that, in mammals, CDS length is the main determinant of the shift in translation, and not UTR length or structure. Our results are of importance for the interpretation of for instance the long-standing ribosome concentration hypothesis [58], which models how absolute differences in ribosome availability can simultaneously reduce or increase the efficiency of mRNA translation depending on mRNA intrinsic translation rates. We show that these mRNA intrinsic differences in initiation rates are, to a large extent, not driven by classical *cis* effector elements such as uORFs and 5′ UTR structure, but primarily by CDS length and hence the frequency of ribosome reinitiation.

Based on this evidence, we propose a unified model that lays the foundation of these specific translational phenotypes. For this model, it is important to know that length-dependent differences in the efficiency of translation are present at baseline in the translatomes of all species [27–29]. These differences are directly connected to the rate of translation initiation [27–29] and can be explained by varying rates of translation reinitiation [28]. As a single round of translation at a short CDS takes less time to complete, reinitiation rates are higher, which ultimately yields more protein. Hence, when translation initiation rates are reduced, this does not necessarily decrease the efficiency of translation reinitiation, as both subunits have already been recruited and properly assembled once, as accurately modelled by Rogers et al. [28]. It does make mRNAs more dependent on effective and frequent reinitiation for their translational output, thereby enhancing a pre-existing length-dependent imbalance in TE—which is exactly what we observed in the rat hearts that carried the SHR genotype at the Chr. 3p teQTL (Fig. 4A, B). We postulate that the loss of several translation initiation factors, such as eIF4A or eIF4B, can similarly affect the efficiency of translation initiation, resulting in translational profiles with length effects nearly identical to the ones observed in our rat models. In the opposite scenario, factors that influence closed loop formation (e.g., Asc1/RACK1 or eIF4G1) would reduce the ability of ribosomes to reinitiate when mutated, with limited impact on first round translation initiation. In comparison to the wild type situation, where a length dependency is generally present, hampered reinitiation results in a length-dependent shift in TE with negative consequences for genes with short CDSs (where reinitiation is frequent), as opposed to a lesser penalty on longer CDSs that generally depend more on canonical translation initiation.

Because of sequencing data normalization, all shifts in TE between control and mutant samples are relative. This means that they could go accompanied by overall absolute changes in translational output (i.e., a general reduction in translation), which would be “quenched” during the data normalization steps. Our results reveal gene-specific shifts in TE relative to other genes within the sample or genetic background investigated. Such changes are particularly relevant for our understanding of how the translation machinery controls gene expression regulation, or how imbalances in translation rates can have consequences for the composition stoichiometry of protein complexes, as we demonstrate for the cardiac sarcomere. This thus far largely overlooked consequence of translational deficiencies appears to be conserved from yeast to humans and could be an important mediator of the molecular changes that connect common ribosomopathies with specific clinical symptoms in a tissue-specific manner [91].

## Conclusions

Our work shows that naturally occurring genetic variation can induce a complex, translation-driven molecular mechanism that globally reforms mammalian tissue gene expression. Distant genetic control of mRNA translation is frequent and contributes significantly to interindividual phenotypic variability, coordinated by multiple master regulatory loci that each regulate the TE of multiple genes. We anticipate that adaptation of gene expression regulation through mRNA translation is crucial for tissues developing complex phenotypic traits. We highlight a single genetic locus that influences TE in a protein length-dependent fashion, as a result of a deficiency in mRNA translation. We show that translatome-wide and length-dependent shifts in TE are more common for translation machinery mutants—including those that cause common ribosomopathies—and present a simplified, CDS length-dependent model that could explain the directionality and extent of this effect.

## Methods

### Animal models

Six-week-old male HXB/BXH RI rats (n = 30; left ventricle and liver), congenic SHR.BN-D3Rat108/D3Rat56 rats (SHR.BN-(3L); n = 5; left ventricle), congenic SHR.BN-D3Rat108/D3Rat124 rats (SHR.BN-(3S); n = 5; left ventricle), transgenic SHR/Ola-Tg rats expressing *Endog* (n = 5; left ventricle), and wild type SHR/Ola rats carrying an *Endog* LoF mutation (n = 5; left ventricle) were housed, bred, and fed ad libitum with a natural diet (Altromin 1314) in an air-conditioned animal facility at the Czech Academy of Sciences, Prague, Czech Republic. The congenic rat lines were designed as follows (as described in [20]): For (SHR.BN-D3Rat108/D3Rat56 or “SHR.BN-(3L)”), a longer genomic BN fragment (Chr3 0–60 Mb) replaces the entire Chr. 3p teQTL in an otherwise fully SHR/Ola genetic background. For (SHR.BN-D3Rat108/D3Rat124 or “SHR.BN(3S)”), only a shorter fragment (Chr3 11.2–60 Mb) adjacent to, but not overlapping, the identified teQTL is replaced with a BN fragment. Transgenic SHR/Ola-Tg(CMV-*Endog*)136 strain (hereafter referred to as the SHR-*Endog* transgenic) was derived by microinjecting fertilized eggs with a mix of the Sleeping Beauty construct containing *Endog* cDNA of BN origin under control of the universal EF-1α promoter and mRNA of the SB100X transposase. Transgenic rats were detected using PCR with the following primers: *Endog*-F 5′-CGA CAC CTT CTA CCT GAG CA-3′ and *Endog*-R 5′-GGC CCT GTG CAG ACA TAA AC-3′. The rats were killed by cervical dislocation without prior anesthesia.

The Endog KO mouse was derived from a C57BL/6J background and provided by Dr. Michael Lieber, University of Southern California, LA, CA, USA [92]. From the provided founder animals, a colony was established and actively maintained for multiple years within the lab of Daniel Sanchis (Institut de Recerca Biomedica de Lleida, Spain) [20]. The investigation with the Endog KO line was approved by the Experimental Animal Ethic Committee of the University of Lleida and conforms to the Guide for the Care and Use of Laboratory Animals, 8th Edition, published in 2011 by the US National Institutes of Health. The 6-week-old male mice used for Ribo-seq and RNA-seq experiments were housed in Tecniplast GM500 cages (391 × 199 × 160 mm) never exceeding 5 adults / cage. Animals were anesthetized with a lethal dose of inhaled isofluorane and decapitated within the facility by expert staff.

### Generating a genotype map of the HXB/BXH panel

To refine an existing [3, 30] genotype map of the HXB/BXH panel and convert this map to the latest rat genome assembly (rn6), we genotyped the 30 HXB/BXH recombinant inbred panel lines using a custom-designed Affymetrix RATDIV single-nucleotide polymorphism (SNP) Array at 805,399 variable genetic positions, as described previously [93]. In short, genotyping was performed according to the Affymetrix SNP chip 6.0 protocol using 250 ng (RNase A-treated) genomic DNA, isolated from rat liver tissue and digested with StyI and NspI, respectively. Genotypes were called and high-quality markers were selected from the 805,399 genotyped SNPs. For this, the original 25-mer Affymetrix probes were first remapped to the latest Ensembl rat genome build (Rnor 6.0) [94] using BLAST [95], requiring the wild type or variant probe to map uniquely within the entire rat genome (as described previously in [93]). We furthermore excluded (i) SNPs within 13 base pairs of an indel, (ii) missing or heterozygous variant calls, (iii) monomorphic markers, and (iv) SNPs with a call rate lower than 0.99. The resulting genotype calls could be collapsed into 2957 genotype blocks, or strain distribution patterns (SDPs), with an average size of 0.75 Mb. Collapsing genotypes into SDPs increased the power for downstream QTL mapping, as not every SNP had to be tested individually. An SDP changed to a next SDP as soon as one of the 30 lines consistently switched genotypes. As some SDPs can occur more than once in the genome, e.g., by chance or because of genotyping or genome assembly errors, we merged such SDPs into a single, globally uniquely occurring SDP, while preserving positional information. Subsequently, we merged identical SDPs if separated by a single alternating SDP (e.g., due to a SNP genotyping error). This results in a set of 1685 unique SDPs that we subsequently used for QTL mapping (Additional file 6: Table S5).

### Ribosome profiling of heart and liver tissue

For ribosome profiling and mRNA-seq, snap-frozen and powdered tissue was obtained from the animals described in the “Animal models” section. For all samples except for the transgenic *Endog* rats and the *Endog* knockout mice (see below), ribosome profiling was performed using the TruSeq Ribo Profile (Mammalian) Library Prep Kit (Illumina, San Diego, CA, USA), according to a TruSeq Ribo Profile protocol optimized for use on tissue material, as described previously [31, 96]. In short, ± 50–100 mg powdered tissue was lysed for 10 min on ice in 1 mL lysis buffer consisting of 1 × TruSeq Ribo Profile mammalian polysome buffer, 1% Triton X-100, 0.1% NP-40, 1 mM dithiothreitol, 10 U ml^− 1^ DNase I, cycloheximide (0.1 mg ml^− 1^), and nuclease-free H_2_O. Using immediate repeated pipetting and multiple passes through a syringe with a 21G needle, we dissociated tissue clumps to create a homogenous lysate that facilitates quick and equal lysis of the tissue powder. Samples were next centrifuged at 20,000*g* for 10 min at 4 °C to pellet cell and tissue debris. Per sample, 400–800 μl of lysate was further processed according to the TruSeq Ribo Profile (Mammalian) Reference Guide with the additional modification of 8% PAGE selection directly after PCR amplification of the final library. For all samples, ribosome profiling library size distributions were checked on the Bioanalyzer 2100 using a high-sensitivity DNA assay (Agilent; 5067-4626), multiplexed, and sequenced on an Illumina HiSeq 2500 producing single end 1 × 51 nt reads. HXB/BXH RI panel samples were always processed in large batches of maximum 30 samples to avoid a sample processing bias.

For heart tissue of transgenic and wild type SHR/Ola rats, as well as *Endog* knockout and wild type C57BL/6 mice, a slightly modified procedure was used due to the termination of the TruSeq RiboProfile kit production by Illumina. The isolation of ribosome footprints is identical to the procedure with the TruSeq kit and as described in [31], except for the use of 7.5 μL Ambion RNase 1 (Thermo Fisher Scientific AM2295; 100 U/μL). Following footprint isolation and PAGE purification, footprints were phosphorylated (NEB T4 PNK; New England Biolabs M0201) and used as input for small RNA library prep using the NEXTflex Small RNA-Seq Kit v3 (Bioo Scientific - PerkinElmer NOVA-5132-06). Libraries were prepared according to the manufacturer’s instructions (V19.01), size-selected on 8% PAGE gels (Thermo Fisher Scientific EC6215BOX), and quality checked on a Bioanalyzer 2100 (high sensitivity DNA assay; Agilent; 5067-4626). Libraries displayed an average size of 157 bp and were sequenced in a multiplexed manner averaging 4 samples per lane on an Illumina HiSeq 4000. Downstream Ribo-seq data QC shows identical read quality, library complexity, and footprint periodicity as libraries generated by Illumina’s TruSeq RiboProfile procedure.

### Replicate HXB/BXH Ribo-seq experiments

On average, each genomic locus within the HXB/BXH RI panel is shared by 15 animals, as all 30 RI lines are a homozygous mixture of 2 genetic backgrounds (BN-*Lx* and SHR/Ola). To assess the biological variability across individual animals of each HXB/BXH RI line, we performed replicate Ribo-seq experiments on liver tissue of 3 animals (i.e., biological replicates) for 2 of the 30 RI lines: BXH12 and BXH13. For each, we find Pearson correlations > 0.99 across biological replicates, reassuring the high quality of our data and reproducibility of the library preparation and sequencing approach (Additional file 1: Figure S1C).

### mRNA-seq and totRNA-seq

For mRNA-seq and totRNA-seq, total RNA was isolated using TRIzol Reagent (Invitrogen; 15596018) using 5–10 mg rat and mouse tissue of the exact same powdered tissue samples (from the exact same animals) used for Ribo-seq. RNA was DNase treated and purified using the RNA Clean & Concentrator™-25 kit (Zymo Research; R1018). RIN scores were measured on a BioAnalyzer 2100 using the RNA 6000 Nano assay (Agilent; 5067-1511). Poly(A)-purified mRNA-seq libraries or ribosomal RNA-depleted totRNA-seq libraries were generated from the same sample of high-quality RNA (average RNA integrity number (RIN) for HXB/BXH rats of 9.1 (Additional file 1: Figure S1A). RNA-seq library preparation was performed according to the TruSeq Stranded mRNA or total RNA Reference Guide, using 500 ng of total RNA as input. Libraries were multiplexed and sequenced on an Illumina HiSeq 2500 or 4000 producing paired-end 2 × 101 nt reads.

### Polysome profiling of congenic rat hearts

Powdered left ventricular heart tissue (3 replicates per congenic line) was lysed in polysome lysis buffer composed of 20 mM Hepes pH 7.5, 5 mM MgCl2, 300 mM KCl, 2 mM DTT, 100 μg/mL cycloheximide, 0.2% NP-40, and 40 U/μl RNAseOut (Invitrogen). Following a 30-min incubation at 4 °C in rotation, the lysed tissue samples were centrifugated for 15 min at 20,000×*g* at 4 °C. An aliquot of the lysate was used to quantify total RNA concentration using the Direct-zol RNA kit (R2051; Zymo, USA) according to the manufacturer’s instructions. From the clear supernatants of the lysates, 15 μg of total RNA was loaded onto 10–50% linear sucrose gradients prepared in polysome buffer (20 mM Hepes pH 7.5, 5 mM MgCl_2_ and 300 mM KCl, 2 mM DTT), and centrifuged at 32,000 rpm (129,311×*g*) (SW40Ti rotor, Beckman) for 177 min at 4 °C. Sucrose gradient fractions were separated using a Biocomp Piston gradient fractionation system associated to a Biorad fraction collector (Biorad model 2110 Fraction Collector) into 42 fractions of 300 μl each, and the absorbance was monitored at 254 nm with an ultraviolet absorbance detector (Biorad model EM-1 Econo UV monitor) to record the polysome profile. Fractions corresponding to the monosomes, light, medium, and heavy polysomes were pooled separately. RNA was extracted with 3× volumes of TriFast-FL (VWR, USA) and purified using Direct-zol RNA kit (Zymo, USA) according to the manufacturer’s instructions. RNA was DNase treated and purified using the RNA Clean & Concentrator™-25 kit (Zymo Research; R1018). RIN scores were measured on a BioAnalyzer 2100 using the RNA 6000 Nano assay (Agilent; 5067-1511). Ribosomal RNA-depleted totRNA-seq libraries were generated from high-quality RNA (Additional file 2: Table S1). RNA-seq library preparation was performed according to the TruSeq Stranded total RNA Reference Guide, using 200 ng of total RNA as input. Libraries were multiplexed and sequenced on an Illumina HiSeq 4000 producing paired 2 × 78 nt reads.

### Sequencing data alignment

Prior to mapping, ribosome profiling reads were clipped for residual adapter sequences and filtered for mitochondrial, ribosomal RNA, and tRNA sequences. Next, we trimmed the 2 × 101 nt mRNA-seq reads to 29-mers (matching Ribo-seq footprint lengths, which peak at 28-29 nt) and processed those mRNA reads exactly the same as the ribosome profiling data, in order to avoid a downstream mapping or quantification bias due to read length or filtering. For mapping of the HXB/BXH rat RI panel data, we first used Tophat2 v2.1.0 [97] to align the full-length 2 × 101 nt mRNA-seq against the rat reference genome (*Rattus Norvegicus* rn6, Ensembl release 82), in order to obtain all splicing events naturally occurring in heart and liver tissue. Next, all 29-mer trimmed mRNA and ribosome profiling data were mapped using the splice junction information gathered from the alignment of the full-length mRNA-Seq reads. TopHat2 was used for the initial sequencing data alignment and splice junction determination of the HXB/BXH data analysis, as at the time this project was initiated current state-of-the-art alignment tools were not yet available. Sequencing data was aligned to the reference genome, and not to reconstructed SNP-infused genomes, because the number of allowed mismatches per 29-mer (2 mismatches) suffices to overcome a mapping bias caused by SHR-specific SNPs. We tested this reasoning extensively by aligning replicate trimmed mRNA-seq and Ribo-seq data of SHR/Ola animals (5 replicates) [96] to the BN reference genome or to an SHR/Ola SNP-infused genome. Moreover, we detected no significantly differentially expressed genes, i.e., genes for which the expression change could be attributed to a mapping bias driven by local genetic variation. On average, for the HXB/BXH Ribo-seq data, we can uniquely align 27.8 M Ribo-seq reads for left ventricular tissue samples and 41.5 M Ribo-seq reads for liver tissue samples, equaling between 71 and 87% of the total number of sequenced reads used for mapping.

For Ribo-seq and RNA-seq data obtained from congenic rats, transgenic rats, knockout mice, and polysome fractionation experiments, sequencing alignment strategies were identical to described above, but using STAR 2-pass v2.7.1a [98] instead of TopHat2 to improve mapping accuracy and speed. Mice data was mapped to the *Mus Musculus* reference genome mm10, Ensembl release 85. We used STAR to align the previous datasets mapped with Tophat2 and we found Pearson correlations > 0.99 across both methods, supporting the reproducibility of the data regardless of the mapping algorithm. Data QC of all Ribo-seq libraries was performed using Ribo-seQC v1.1 [99].

### Identifying translated open reading frames

To define the set of translated genes in rat heart and liver, we used RiboTaper v1.3 [100] with standard settings to detect open reading frames that display the characteristic 3-nt codon movement of actively translating ribosomes. For each sample, we selected only the read lengths for which at least 70% of the reads matched the primary ORF in a meta-gene analysis. This results in the inclusion of footprints of the most prominent read lengths: 28 and 29 nucleotides. The final list of translation events was stringently filtered requiring the translated gene to have an average mRNA-seq RPKM ≥ 1 and be detected as translated by RiboTaper in at least 10 out of 30 HXB/BXH RI lines. We did not only retain canonical translation events, but also translated short ORFs (sORFs) detected in long noncoding RNAs (lncRNAs), or upstream ORFs (uORFs) positioned in front of primary ORFs of annotated protein-coding genes. LncRNA sORFs were required to not show sense and in-frame overlap with annotated protein-coding genes. We categorically grouped noncoding genes with antisense, lincRNA, and processed transcript biotypes as long noncoding RNAs (lncRNAs), if they matched specific filtering criteria described previously [31]. Upstream ORFs encompass both independently located (non-overlapping) and primary ORF-overlapping translation events. Primary ORF-overlapping uORFs were distinguished from in frame, 5′ extensions of the primary ORF requiring each overlapping uORF to have a translation start site before the start of the canonical CDS, to end within the canonical CDS (prior to the annotated termination codon) and to be translated in a different frame than the primary ORF, i.e., to produce a different peptide. We combined both types of uORFs into a single uORF category as we detect no differential impact of each uORF category on the primary ORF TE, in accordance with previous work [31]. For the visualization of P-site tracks (Additional file 1: Figure S4E), we used plots generated by Ribo-seQC [99].

### Quantifying mRNA expression and translation

Gene- or feature-specific expression quantification was restricted to annotated and identified translated (coding) sequence and performed using HTSeq v0.9.1 [101] with default parameters. For quantifying ribosome association in small and long noncoding RNAs, i.e., genes without annotated coding sequences (CDSs), we additionally ran HTSeq on exonic gene regions. For quantification of the *Ttn* gene, which codes for the longest protein existing in mammals, we used a custom annotation [31, 102] as *Ttn* is not annotated in the current rat gene annotation. For this reason, *Ttn* was initially not included in the QTL mapping analyses, but later on added to define the effect of its length on *Ttn*’s translational efficiency. Moreover, we masked one of the two identical SURF cluster regions in the rat genome (chr3:4,861,753-4,876,317 was masked and chr3:5,459,480-5,459,627 was included), as both regions shared 100% of nucleotide identity and the six expressed *SURF* genes could not be unambiguously quantified. Since 406 snoRNAs have paralogs with 100% of sequence identity and unique counts cannot be unambiguously assigned to these sequences, these RNAs were not considered for quantification. In summary, we thus used (i) uniquely mapping CDS-centric counts for mRNA and translational efficiency quantifications, and (ii) uniquely mapping exonic counts for noncoding RNA quantifications (e.g., *SNORA48*) after excluding snoRNAs clusters sharing 100% of sequence similarity.

The mRNA-seq and Ribo-seq count data was normalized using a joint normalization procedure (estimateSizeFactorsForMatrix; DESeq2 v1.26.0 [103]) as suggested previously [104]. This allows for the determination of size factors for both datasets in a joint manner, as both count matrices follow the same distribution. This is crucial for the comparability of the two sequencing-based measures of gene expression, which for instance becomes important for calculating a gene’s translational efficiency (TE). The TE of a gene can be calculated by taking the ratio of Ribo-seq reads over mRNA-seq reads [22], or, when biological replicates are available, calculated via specialized DESeq2-based tools [104–106]. As we here require sample-specific TE values for downstream genetic association testing with QTL mapping, we regress out the measured mRNA-seq expression from the Ribo-seq expression levels using a linear model. This allows us to derive residuals for each sample-gene pair, that we subsequently subject to QTL mapping. Thus, the TE refers to the residuals of the linear model: resid (lm (normalized_Ribo-seq_read_counts ~ normalized_mRNA-seq_read_counts)). The main advantage of TE values obtained with this calculation is that we retain a quantitative range suitable for QTL mapping, which would not be the case for ratio-based TEs.

### Pairwise association testing using Matrix eQTL

In order to understand the impact of genetic variants on gene expression regulation, we performed quantitative trait locus (QTL) mapping using the linear regression model-based Matrix eQTL v2.1.1 [107]. For association testing, non-unique SDPs are grouped and associations of surrounding SDPs are considered when defining the correct SDP location, thereby avoiding falsely assigned distant QTLs because of misplaced contigs in the rn6 rat genome assembly. For this, we reasoned that true associations are likely visible in surrounding SDPs, as genotype changes between two neighboring SDPs are usually gradual, and only a statistically unlikely multitude of recombination events between two neighboring SDPs would fully quench the detected association. After each association is assigned to the correct SDP, we performed a Benjamini-Hochberg correction on local and distant associations separately. Subsequently, we performed permutation testing to determine the significance of local and distant associations, by deriving the distribution of test statistics under the null hypothesis that there is no association. We therefore randomized all samples in the gene expression matrix and performed 10,000 runs of Matrix eQTL on the original genotype matrix. A significant association was defined as having an empirical p value ≤ 0.0015 (less than 15 more extreme p values in 10,000 permutations). For all types of QTLs tested in this study (eQTLs, riboQTLs, teQTLs, and uORF-QTLs), the same association settings and filtering criteria are applied.

A QTL is defined as “local” when it locates within the SDP block of the gene locus for which the association was detected. Similarly, a distant QTL is defined as a trait-associated locus when it is located on a chromosome different from the one that hosts the associated gene. In order to evaluate the presence of cross-mappability artifacts when identifying distant QTLs, we adapted a published method [108] and identified pairs of sequences with shared 29-bp k-mer sequences that are susceptible to be cross-mapped, allowing a maximum of 2 mismatches. In the heart, only 1 gene (ENSRNOG00000054609) with a distant QTL was cross-mappable with a *cis*-gene (ENSRNOG00000019925) in the same SDP. This distant QTL was therefore filtered out. Moreover, no cross-mappable genes with distant QTLs were found in liver. Therefore, our analysis reassured that cross-mapping did not affect the detection of distant QTLs. Throughout the manuscript, QTL numbers reported are gene-centric, i.e., if for instance two neighboring SDPs show significant association with the same gene, a single association is counted. When a given gene associates with both local and distant SDPs, these associations are reported separately. We additionally tested all available technical covariates for a potential impact on our results. These included (i) date of tissue processing, (ii) individual who prepared the libraries, (iii) RIN of the sample, (iv) library concentration (after PCR amplification), (v) date of library PCR, and (vi) sequencing batch. None of these technical covariates showed a significant impact on our data (ANOVA p values between 0.11 and 0.97; using the first PC1 that describes 50–80% of the variance in the data). In addition, we also tested additional confounding factors for a possible impact on our results. We calculated the level of relatedness by assessing the covariance of the genotypes across all 30 recombinant inbred lines (average covariance of 0.506; Additional file 1: Figure S1I + J). Additionally, we ran fastSTRUCTURE [109] to investigate the population structure of our HXB/BXH rat RI panel, identifying five distinct subpopulations of 3–12 individuals defined by different SDP allele frequencies. We used these allele frequencies to estimate the fixation index (F_st_): F_st_ = 1 − (Hs / H_T_), where Hs is the average expected heterozygosity within subpopulations and H_T_ corresponds to the expected heterozygosity of the total population. A fixation index value of 0 indicates no differentiation between the defined subpopulations, whereas a value of 1 corresponds to complete differentiation [110]. In this case, the average fixation index for the panel was 0.203, suggesting a limited effect of the population structure in the observed genetic differences across individuals. Next, we used lme4qtl [111] to build a linear mixed model considering both relatedness and population structure, and we estimated the robustness of the identified QTLs by statistically comparing the linear mixed model with a null model where the genetic effects were not included. Reassuringly, 91.88–96.17% of *cis* and 80.00–100.00% of *trans* QTLs displayed significance in the linear mixed model (adjusted ANOVA p values < 0.0001). Additionally, we tested if QTL effects were largely driven by the presence of hidden surrogate variables. First, we evaluated the specific effect of hidden confounders in the matrix eQTL calculations by using the “sva” R package [112]. Correlation of original and corrected effect sizes for heart local QTLs confirmed that these covariates did not significantly impact our data (Pearson correlation > 0.99; Additional file 1: Figure S1K). Noteworthy, for distant teQTLs, the correlations corrected by surrogate variable were less significant (Pearson correlation 0.79–0.99). Second, we added the top three, five, and ten predicted covariates automatically predicted by the PEER package [113] to the linear mixed model, finding a similar fraction of QTLs with robust significance regardless of the inclusion of PEER covariates (90.63–96.60% of *cis* and 76.47–100.00% of *trans* QTLs). In large cohorts such as the Human GTEx Project, data is often collected from different sources and hidden covariates can explain a large fraction of the total variance in *trans* QTLs, stressing the need of aggressive corrections of potential confounders, which are common practice [114]. However, including hidden covariates did not significantly affect the results of our data as these added covariates explained a small fraction of the total variance. Moreover, correcting for hidden surrogate variables can negatively impact the detection of *trans* loci that associate with multiple genes [113]. Consequently, because of the robustness of our results when correcting for known and unknown effects on the identified set of QTLs, we decided to use the full set of detected QTLs for subsequent analyses.

All detected significant association results, including additional significance values for QTLs after correcting for relatedness, population structure, and hidden covariates, are reported in Additional file 3: Table S2 (eQTLs, riboQTLs and teQTLs).

### Detection of tissue-specific and recurrent QTLs

Gene expression can be regulated in a highly tissue- and cell-type-specific manner and genetic effects on mRNA expression can similarly be both specific to, or shared amongst, tissues or cell types [8, 32]. Nevertheless, the difference in QTL significance between tissues can represent an artifact because of the presence of false negatives in one of the tissues. Hence, we estimated the statistical power to replicate QTLs across tissues and traits within the HXB/BXH panel adapting a method previously applied to RI lines [115]. For this, we calculated narrow-sense trait heritabilities (*h*^2^_trait_) using the formula reported by Bottolo et al. [116], based on the method of Hegmann and Possidente [117]: *h*^2^_trait_ = 0.5*V*_A_/(0.5*V*_A_ + *V*_E_), where *V*_A_ is the additive genetic component, representing the variance of the strain means, and *V*_E_ is the environmental component, representing the average variance across all strains. We estimated both components using the two replicated lines (BXH12 and BXH13, see “Replicate HXB/BXH Ribo-seq experiments”). The calculated average heritability was 0.443 for all expressed genes and 0.506 for the set of genes significantly associated to QTLs (Additional file 1: Figure S2D).

We estimated a power of 1 for standardized effect sizes above 0.7, which corresponds to the median effect size for the whole set of tissue-specific QTLs. The estimated power was ~ 0.7 for QTLs with standardized effect sizes lower than 0.55 (percentile 5th of the distribution of QTL effect sizes). Hence, only a very small fraction of low effect QTLs are expected to display tissue-specific significance because of undetected false negatives. In these calculations, standardized effect size estimates are fractions that represent differences in mean values of expression between homozygotes as a proportion of the total genetic variance. These values were calculated by running the R function “VarProp” [111] on the previously generated linear mixed models.

Considering only genes expressed in both tissues, both eQTLs and teQTLs show limited recurrence in QTL detection, indicative of high tissue specificity. Even though 83% of genes with cardiac eQTLs (605 out of 726) and 66% of genes with liver eQTLs (248 out of 377) are expressed in both tissues, we could only detect the same eQTL for 126 of these (17%). Similarly, the vast majority genes with teQTLs are expressed in both tissues (88% and 100% in heart and liver, respectively), though only a small fraction of teQTLs (n = 20; 9%) was independently detected in both. Moreover, tissue-specific QTLs exhibited a stronger effect size and missed associations manifested strongly reduced effect sizes, while the distribution of effect sizes remained constant in shared QTLs across both tissues (Additional file 1: Figure S2B). All but one of these recurrent eQTLs and teQTLs result from local associations (Additional file 3: Table S2), indicating strong enrichment of recurrent local over distant QTLs. This is in line with previous observations across human tissues [8, 32] and, in our study, likely influenced by the higher detection sensitivity for local over distant QTLs. A single distant eQTL for *Tmcc2* forms the exception being regulated in *trans* in both tissues (Additional file 3: Table S2). Although isoform-specific expression regulation of human *TMCC2*, driven by local changes in chromatin dynamics, was previously shown to be of biological importance [118], its distant control was not yet known.

### Finding causal variants for local teQTLs

To identify potential causal variants underlying teQTLs, we infused our genotype maps with known SHR/Ola- and BN-*Lx*-specific indels and SNVs that were previously identified through whole-genome sequencing [16, 17, 119]. Among all genes with a local QTL (either eQTL, riboQTL, or teQTL; Additional file 3: Table S2), we detect only 8 coding sequence variants with a predicted deleterious consequence resulting in one stop gain, one essential splice-site mutation, and six missense mutations [120, 121]. Of these, only a single missense variant in the *Lss* gene is associated with TE in the heart (teQTL p_adj_ = 0.0014; Additional file 3: Table S2). We find no variants altering the local translation initiation context or Kozak sequence—a previously proposed frequent cause of local teQTLs [10].

### Detecting distant QTL hotspots with HESS

HESS [42] is a generic Bayesian variable selection approach, associated with an evolutionary stochastic search algorithm [122], and developed to tackle the challenging integrative task of linking parallel high-dimensional multivariate regressions in a computationally efficient way. When *q* genes are predicted by the same set *p* of SNPs, in HESS the prior probability of association between gene *k* (*k* = 1,…,*q*) and SNP *j* (*j* = 1,…,*p*) is decomposed into its marginal effects, i.e., *π*_*kj*_ = *π*_*k*_ × *ρ*_*j*_, *π*_*kj*_ ∈ [0, 1]. In this formulation, *ρ*_*j*_ ≥ 0 captures the relative “propensity” for SNP *j* to influence several genes at the same time. The SNP specific “propensity” *ρ*_*j*_ inflates/deflates the probability *π*_*k*_ of selecting any SNP to be associated with gene *k* in a multiplicative fashion, i.e., the baseline risk for gene *k* to be associated to any SNP is increased/decreased by the “propensity” of SNP *j* to be a key regulatory marker or “hotspot.” For each gene *k*, the a priori baseline risk and the corresponding level of sparsity are controlled through a suitable choice of the hyper-parameters of the density *p*(*π*_*k*_). We ran R2HESS v1.0.1 [41] with default parameters. The marginal posterior probability of inclusion ${\hat{\gamma}}_{kj}=\mathrm{E}\left({\gamma}_{kj}|\boldsymbol{Y},{\gamma}_{\backslash kj}\right)$ indicates the strength of association between gene *k* and SNP *j* after observing the data ***Y*,** and it is calculated as the number of times a particular gene-SNP pair has been selected. Significant gene-SNP associations were declared using a non-parametric FDR approach, where a mixture model of two beta densities was chosen to model the null *H*_0_ and the alternative *H*_1_ distributions. We ran the Expectation-Maximization algorithm [123] on ${\hat{\gamma}}_{kj}$ (*k* = 1,…,*q, j* = 1,…,*p*) to estimate the parameters of mixture model and, for a fixed FDR level, we calculated the optimal cut-off point on *t* such that the estimated FDR is not greater that the desired one. Finally, the proportion of genes associated with each SNP is defined as the average number of genes that are significantly predicted by each SNP. This measure helps to prioritize SNPs that influence multiple genes at the same time and allows the discovery of so-called regulatory hot spots, i.e., genetic loci that are associated with a large number of mRNAs.

### Western blot analysis and quantification

Frozen left ventricle tissues from congenic rats (SHR.BN-(3S) and SHR.BN-(3L)) were lysed in ice-cold modified RIPA buffer (150 mM NaCl, 50 mM Tris HCL pH 7.4, 1% Triton X-100, 0.5% sodium deoxycholate, 0.1% SDS, 5 mM EDTA, and 2 mM EDTA) containing protease (cOmplete™, EDTA-free Protease Inhibitor Cocktail) and phosphatase (PhosSTOP) inhibitors as described in [66]. After incubation on ice for 30 min, samples were centrifuged at 20,000*g* for 15 min at 4 °C and supernatants were transferred to new pre-chilled tubes. Proteins were denatured for 10 min at 70 °C in NuPAGE LDS Sample Buffer (× 4) (Invitrogen; NP0007) and NuPAGE Sample Reducing Agent (× 10) (Invitrogen; NP0009) and separated on NuPAGE 4-12% Bis-Tris Protein Gels (Invitrogen; NP0343BOX) for 30 min in MES buffer (Invitrogen; NP0002) at 200 V. Gels were blotted on PVDF membranes (Immobilon-PSQ Membrane, Merck Millipore; ISEQ00010), and membranes were stained with the following primary antibodies: p-IRE1α Ser724 (NB100-2323, Novus Biological), IRE1α (3294, Cell Signaling Technology), XBP1s (83418S, Cell Signaling Technology), GAPDH (ab125247, Abcam), HSP60 (12165, Cell Signaling Technology), and TOM20 (42406, Cell Signaling Technology). Protein expression was measured with chemiluminescence and quantified using Image Studio Lite software (version 5.2, LI-COR). Background-subtracted densitometric signals from SHR.BN-(3S) and SHR.BN-(3L) samples were normalized against the loading control (and the unphosphorylated protein form in case of p-IRE1α Ser724) and statistically significant differences between SHR.BN-(3S) and SHR.BN-(3L) samples were determined using unpaired Student’s *t* tests. Uncropped, full-size western blots including ladders can be found in Additional file 1: Figure S6.

### Stoichiometry of the cardiac thin filament

The thin filament is composed of five sarcomere subunits—*Actc1*, *Tpm1*, *Tnnc1*, *Tnnt2*, *Tnni3—*where each unit has a known proportion of 7:1:1:1:1 [44]. So as to study how the production rates of the five thin filament proteins deviate from the compositionally stoichiometric optimal ones in the HXB/BXH RI and the congenic rat samples, we estimated the observed proportions by correcting the DESeq2-normalized counts by CDS length and by gene turnover rate. Gene turnover rates for *Actc1*, *Tpm1*, *Tnnc1*, *Tnnt2*, and *Tnni3* have been previously estimated to be 10.3, 5.3, 3.2, 3.5, and 5.5 days, respectively [45].

### Excluding a technical basis for the length effect

Theoretically, sample-specific gene length biases can artificially induce length-related expression differences that in turn contribute to incorrect enrichment of GO terms related to short (e.g., ribosomal) or long (e.g., ECM) proteins [124]. However, for multiple reasons, we deem it highly unlikely that a technical or analytical bias could be responsible for the length-dependent effect observed in our study. First, the RI lines are all genetic mosaics, and the length dependency is specific for a single locus. Second, the length effect is specific to the heart and absent in liver. Third, data generation, normalization, and statistical analysis are all identical for all sequencing samples analyzed. Fourth, no single documented technical covariate explains any of the variance across samples (e.g., date of tissue processing, library preparation batch, sequencing flow cell, or RNA integrity of the sample; see Additional file 2: Table S1). Fifth, Ribo-seq and polysome fractionation experiments in congenic lines fully reproduce the translation phenotype, indicating a model- and technology-independent effect. Sixth, the effect is absent in RNA-seq data and the correlation with length is stronger for CDS length than for total transcript length. Lastly, previous work on *SNORD24* revealed a highly similar polysome half-mer phenotype accompanied by a length-dependent effect on TE [79].

### Thermodynamic properties of 5′ untranslated regions

5′ UTRs of mRNAs are known to be important regulators of translation. The folding free energy is the difference in free energy between an unfolded and folded state. For a given 5′ UTR, a lower folding free energy corresponds to a more stable secondary structure, and it is associated with low rates of translation initiation [60, 125]. Here, we calculated folding free energies for 5′ UTRs using the RNAfold program (v.2.4.13) from ViennaRNA package [126].

### Reanalysis of translational machinery mutant datasets

We downloaded processed [75, 78–80, 83–85] and raw [82, 86] data from nine Ribo-seq studies that have investigated the consequences of various translational machinery mutants in translation. For each dataset, RNA-seq and Ribo-seq counts were normalized and translational efficiencies were calculated following a similar approach than for the rat datasets (see “Methods”—“Quantifying mRNA expression and translation”). We divided the datasets into wild type and mutant, averaging the number of normalized counts when multiple replicates were available. For each human, mouse, and yeast gene, we calculated the maximum CDS length by extracting all CDSs from Ensembl v.85.

We highlighted ten datasets where the TE calculations were strongly influenced by a (likely technical) length-specific artifact in the RNA-seq data (Pearson’s correlation r > 0.15) that was absent in Ribo-seq data—an effect presumably amplified by length normalization of poly(A)-purified RNA of low integrity [124, 127] (Additional file 1: Figure S5D).

### General remarks on quantification and statistical analyses

The generation of figures and execution of statistical tests were performed using R [128]. GO enrichment analyses were performed using gProfiler2 v0.1.8 [129]. A detailed list of software used for data processing, quantification, and analysis is stated in the respective “Methods” sections. We used DESeq2 v1.26.0 [103] to perform differential gene expression analyses for mRNA-seq and Ribo-seq data. Differentially expressed genes were defined with an FDR ≤ 0.05 and a log2 fold change ≤ 1/1.2 or ≥ 1 × 1.2 for downregulated and upregulated genes respectively. Correlation coefficients between coding sequence (CDS) length and fold changes (FC) in gene expression were based on the Standardized Major Axis (sma) Estimation model (R package “smatr”) [130]. Only CDS with a minimum length of 100 nucleotides and an average number of DESeq2-normalized counts higher than 10 were considered for correlation analyses and plotting. Statistical parameters such as the value of n, mean/median, standard deviation (SD), and significance level are reported in the figures and/or in the figure legends.

## Supplementary Information

**Additional file 1: Figure S1-S6**, related to Figs. 1, 2, 3, 4 - Document with supplemental Figures 1, 2, 3, 4, 5, 6, including figure captions.

**Additional file 2: Table S1**, related to Fig. 1 - Sample information for all sequenced rat and mouse tissue samples, including all open reading frames (ORFs) detected in rat heart and liver.

**Additional file 3: Table S2**, related to Fig. 1 - Table with local and distant QTL mapping results for rat heart and liver. Includes mRNA expression level QTLs (eQTLs), ribosome occupancy QTLs (riboQTLs) and translational efficiency QTLs (teQTLs).

**Additional file 4: Table S3**, related to Fig. 1 - Table with upstream ORFs identified in rat heart and liver and detected uORFs-QTLs.

**Additional file 5: Table S4**, related to Fig. 2 - Table with cardiac QTL hotspots as identified by HESS (see methods).

**Additional file 6: Table S5**. Table with SDP genotypes from the refined genotype map of the HXB/BXH recombinant inbred panel.

**Additional file 7.** Review history.

## Data Availability

The datasets generated and analyzed during the current study are available in the European Nucleotide Archive (ENA) repository under accession number PRJEB38096, http://www.ebi.ac.uk/ena/data/view/PRJEB38096 [131]. This accession includes the raw rat and mouse sequencing data reported in this paper and normalized and ready-to-use sequencing read count matrices. Additional datasets analyzed during the current study are available in the Gene Expression Omnibus (GEO) repository, https://www.ncbi.nlm.nih.gov/geo/query/acc.cgi?acc=GSE106529, https://www.ncbi.nlm.nih.gov/geo/query/acc.cgi?acc=GSE121189, https://www.ncbi.nlm.nih.gov/geo/query/acc.cgi?acc=GSE61753, https://www.ncbi.nlm.nih.gov/geo/query/acc.cgi?acc=GSE66411, https://www.ncbi.nlm.nih.gov/geo/query/acc.cgi?acc=GSE81966, https://www.ncbi.nlm.nih.gov/geo/query/acc.cgi?acc=GSE87614, https://www.ncbi.nlm.nih.gov/geo/query/acc.cgi?acc=GSE89183, https://www.ncbi.nlm.nih.gov/geo/query/acc.cgi?acc=GSE111255, https://www.ncbi.nlm.nih.gov/geo/query/acc.cgi?acc=GSE143626 [75, 78–80, 82–86]. All analyses in this study are performed using published and publicly available analytical tools or software packages, with the precise software version parameters used detailed in the respective “Methods” sections. The code used for the main computational analyses in this study is available at GitHub (https://github.com/jorruior/witte_et_al_2021, [132]) and Zenodo [133] under GNU General Public License. The transgenic SHR/Ola-Tg(CMV-*Endog*)136 rat line with rescued expression of *Endog* was newly established for this study in the lab of Michal Pravenec (Institute of Physiology of the Czech Academy of Sciences, 142 20, Praha 4, Czech Republic) and is available upon request.
