# Supplementary material for: A trans locus causes a ribosomopathy in hypertrophic hearts that affects mRNA translation in a protein length-dependent fashion
Source: Genome Biol. 2021 Jun 28;22:191. doi: 10.1186/s13059-021-02397-w (PMC8240307; doi:10.1186/s13059-021-02397-w)
Supplement: Supplementary file 1 — Additional file 1: Figure S1-S6, related to Figs. 1, 2, 3, 4 - Document with supplemental Figures 1, 2, 3, 4, 5, 6, including figure captions. [file 13059_2021_2397_MOESM1_ESM.pdf]

Additional File 1. Supplemental figures S1-S6: A *trans* locus causes a ribosomopathy in hypertrophic hearts that affects mRNA translation in a protein length-dependent fashion

Franziska Witte[1,2,$], Jorge Ruiz-Orera[1,$], Camilla Ciolli Mattioli[3,4], Susanne Blachut[1], Eleonora Adami[1,5], Jana Felicitas Schulz[1], Valentin Schneider-Lunitz[1], Oliver Hummel[1], Giannino Patone[1], Michael Benedikt Mücke[1,6,7], Jan Šilhavý[8], Matthias Heinig[9], Leonardo Bottolo[10], Daniel Sanchis[11], Martin Vingron[12], Marina Chekulaeva[3], Michal Pravenec[8], Norbert Hubner[1,6,7,13,*], Sebastiaan van Heesch[1,14,*]

[1] Cardiovascular and Metabolic Sciences, Max Delbrück Center for Molecular Medicine in the Helmholtz Association (MDC), 13125 Berlin, Germany

[2] Present address: Research and Development, Pharmaceuticals, Bayer AG, 13353 Berlin, Germany

[3] Berlin Institute for Medical Systems Biology (BIMSB), Max Delbrück Center for Molecular Medicine in the Helmholtz Association (MDC), 10115 Berlin, Germany.

[4] Present address: Department of Biological Regulation, Weizmann Institute of Science, 7610001, Rehovot, Israel.

[5] Present address: Program in Cardiovascular and Metabolic Disorders, Duke-National University of Singapore, Singapore 169857, Singapore.

[6] DZHK (German Centre for Cardiovascular Research), Partner Site Berlin, 13347 Berlin, Germany.

[7] Charité -Universitätsmedizin, 10117 Berlin, Germany.

[8] Institute of Physiology of the Czech Academy of Sciences, 142 20, Praha 4, Czech Republic

[9] Institute of Computational Biology (ICB), HMGU, Ingolstaedter Landstr. 1, 85764 Neuherberg (Munich), Germany; Department of Informatics, Boltzmannstr. 3, 85748 Garching, Technische Universitaet Muenchen (TUM), Munich, Germany.

[10] Department of Medical Genetics, University of Cambridge, Cambridge, CB2 0QQ, UK; The Alan Turing Institute, London, NW1 2DB, UK; MRC Biostatistics Unit, University of Cambridge, Cambridge, CB2 0SR, UK.

[11] Institut de Recerca Biomedica de Lleida (IRBLLEIDA), Universitat de Lleida, Edifici Biomedicina-I. Av. Rovira Roure, 80, 25198 Lleida, Spain

[12] Max Planck Institute for Molecular Genetics, Department of Computational Molecular Biology, 14195 Berlin, Germany.

[13] Berlin Institute of Health (BIH), 10178 Berlin, Germany.

[14] Present address: The Princess Máxima Center for Pediatric Oncology, Utrecht, the Netherlands.

[$] These authors contributed equally

*Correspondence: s.vanheesch@prinsesmaximacentrum.nl and nhuebner@mdc-berlin.de

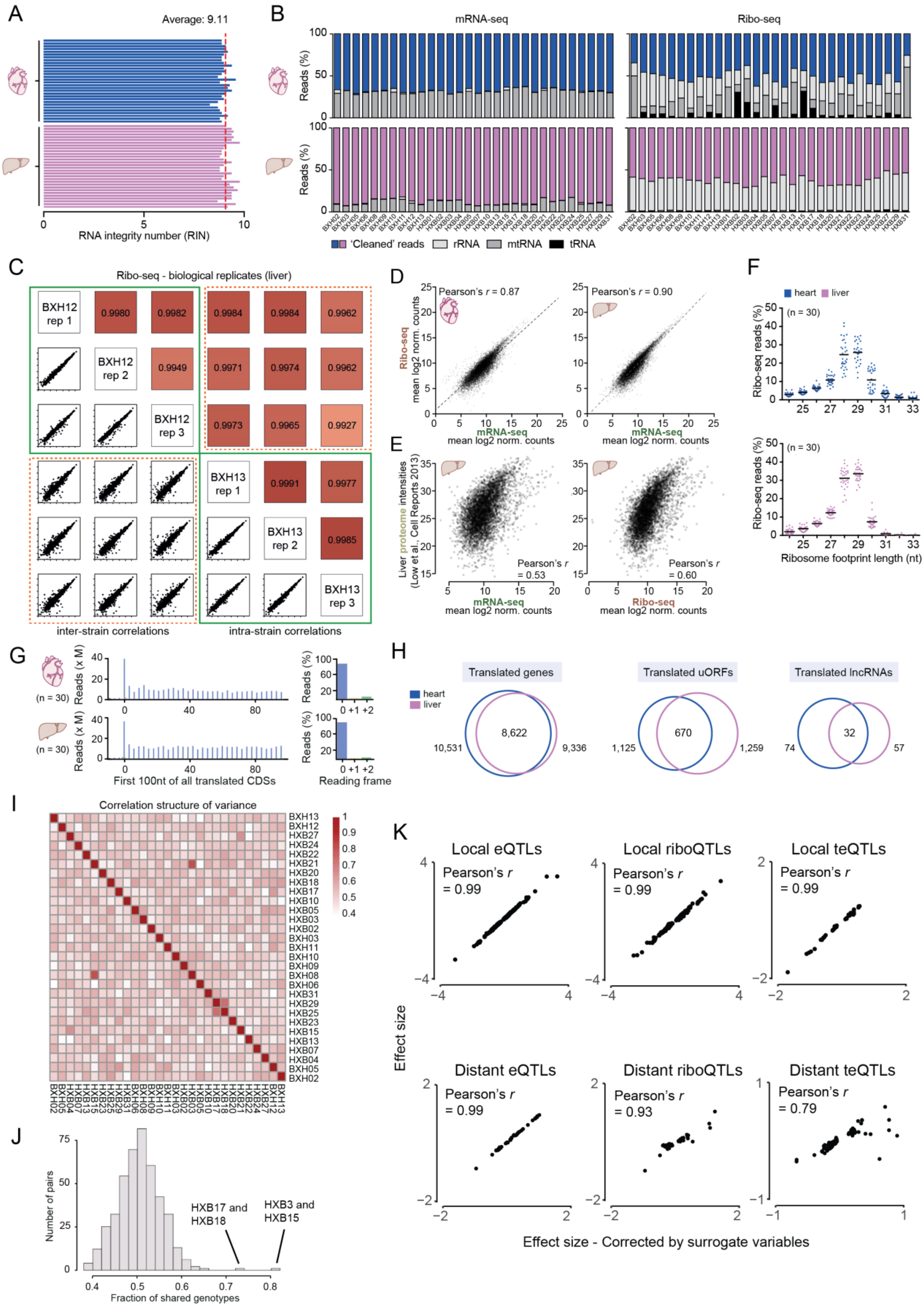
A
Average: 9.11
RNA integrity number (RIN)
B
mRNA-seq
Ribo-seq
Reads (%)
'Cleaned' reads
rRNA
mtRNA
tRNA
C
Ribo-seq - biological replicates (liver)
BXH12 rep 1
BXH12 rep 2
BXH12 rep 3
BXH13 rep 1
BXH13 rep 2
BXH13 rep 3
0.9980
0.9982
0.9984
0.9984
0.9962
0.9949
0.9971
0.9974
0.9962
0.9973
0.9965
0.9927
0.9991
0.9977
0.9985
inter-strain correlations
intra-strain correlations
D
Pearson's r = 0.87
Pearson's r = 0.90
Ribo-seq mean log2 norm. counts
mRNA-seq mean log2 norm. counts
E
Liver proteome intensities (Low et al., Cell Reports 2013)
Pearson's r = 0.53
Pearson's r = 0.60
mRNA-seq mean log2 norm. counts
Ribo-seq mean log2 norm. counts
F
heart
liver
(n = 30)
Ribo-seq reads (%)
Ribosome footprint length (nt)
G
(n = 30)
Reads (x M)
Reads (%)
First 100nt of all translated CDSs
Reading frame
H
heart
liver
Translated genes
10,531
8,622
9,336
Translated uORFs
1,125
670
1,259
Translated lncRNAs
74
32
57
I
Correlation structure of variance
J
Number of pairs
HXB17 and HXB18
HXB3 and HXB15
Fraction of shared genotypes
K
Local eQTLs
Pearson's r = 0.99
Local riboQTLs
Pearson's r = 0.99
Local teQTLs
Pearson's r = 0.99
Distant eQTLs
Pearson's r = 0.99
Distant riboQTLs
Pearson's r = 0.93
Distant teQTLs
Pearson's r = 0.79
Effect size
Effect size - Corrected by surrogate variables

Additional File 1: Figure S1: Quality of sequencing datasets and QTL mapping, related to Figure 1. (**A**) Bar plot with RNA integrity numbers (RIN values) for total RNA isolated from rat heart (top) and liver (bottom). The dashed line indicates the average RIN of 9.11, illustrating the high integrity of the processed tissue samples. (**B**) Stacked bar plots with sequencing read filtering statistics for heart (top) and liver (bottom) tissue. Reads derived from ribosomal RNA (rRNA), mitochondrial RNA (mtRNA) and transfer RNA (tRNA) are removed from the mRNA-seq and Ribo-seq data prior to mapping. This results in a set of 'cleaned reads', which are used as input for mapping and downstream data analyses. (**C**) Correlation analyses and scatter plots for biological replicate Ribo-seq data for 3 replicates of two RI lines (BXH12 and BXH13), illustrating the high technical reproducibility of our Ribo-seq approach across biological replicates. Pearson's r coefficients are also displayed. (**D**) Correlation scatter plots for heart (left) and liver (right) tissue, showing the correlation between mean mRNA-seq and Ribo-seq based quantifications of gene expression. (**E**) Correlation scatter plots between mRNA-seq or Ribo-seq reads and rat liver proteomics data, as quantified using iBAQ values obtained from MaxQuant [1]. Ribo-seq is a slightly better proxy for final protein levels than mRNA-seq (Pearson's r = 0.60 vs 0.53). (**F**) Dot plot showing the ribosome footprint (Ribo-seq read) length distribution across the 30 lines in rat heart (left) and liver (right). (**G**) Venn diagrams with tissue-specific comparisons of all identified translated genes, translated lncRNAs and translated uORFs. (**H**) Bar plot with a meta-analysis of stacked P-sites derived from Ribo-seq reads in heart (top) and liver (bottom) tissue. Blue bars indicate the number (left) and percentage (right) of footprints that precisely match annotated protein-coding gene open reading frames (ORFs; ± 90%). **(I)** Heatmap and **(J)** histogram with correlations of covariances of the genotypes across all 30 recombinant lines. The average level of recombinant inbred relatedness is 0.506. **(K)** Correlation scatter plots for heart QTL effect sizes calculated by standard QTL mapping (Y-axis) and after correcting for surrogate variables (x-axis). This correction can negatively impact the detection of *distant* QTLs that associate with multiple genes. Pearson's r coefficients are also displayed. The high correlation indicates that these covariates did not significantly affect our data and were not included in further analysis.

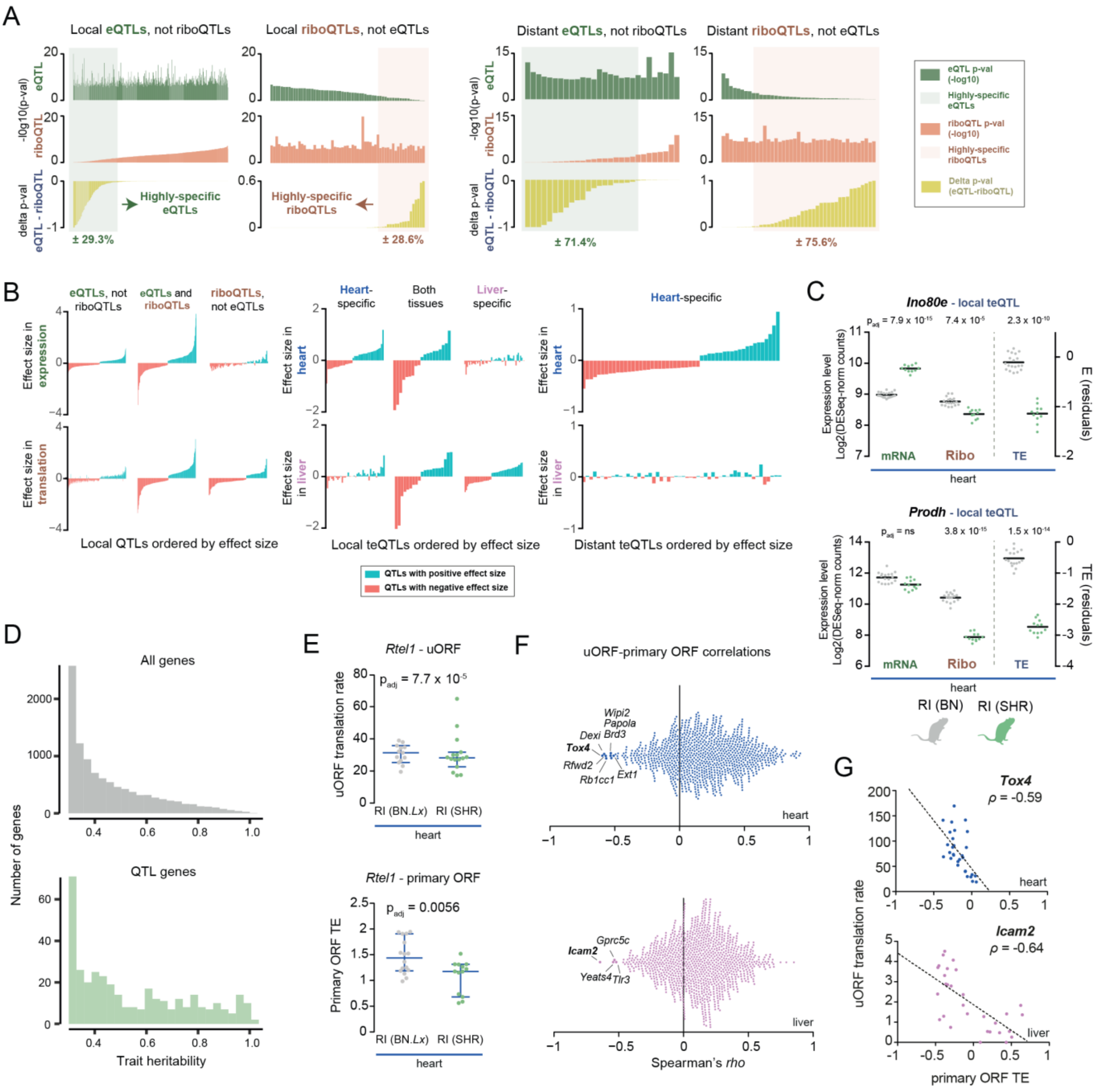

Additional File 1: Figure S2: Identification of translational efficiency QTLs in the HXB/BXH panel, related to Figure 1. (**A**) Bar plots with significance values for detected local (top) and distant (bottom) eQTLs and riboQTLs, sorted by the delta of the p-values for both quantitative traits (bottom track of each panel). (**B**) Bar plots with effect sizes for detected local QTLs by category in heart (left), detected local teQTLs by tissue (medium), and detected distant teQTLs by tissue (right), sorted by effect size. Only three teQTLs were detected in liver, as a result only heart-specific teQTLs are displayed in the bottom plot. Figures A and B illustrates that most eQTLs are prolonged during translation, though they may sometimes near-miss the significance cutoff. Concordant with the teQTL results, this analysis furthermore highlights a highly translation-specific set of QTLs. (**C**) Dot plots with expression values for 2 genes with a highly specific local teQTL in rat hearts. Bars indicate mean values. (**D**) Histograms with trait heritability values estimated for all expressed genes and QTL genes. The average narrow-sense heritability for QTL genes is 0.506, a value higher than for the whole set of expressed genes (0.443). (**E**) *Rtel1* is the only gene for which a local teQTL and uORF-QTL coincide, though both associations occur with similar directionality (lower translation associate with the SHR/Ola genotype). (**F**) Bee swarm dot plots with correlation values (Spearman's *rho*) between the

translation rates of uORFs and the primary ORF TE in heart and liver. Genes with uORFs that show a strong negative correlation with primary ORF translation are highlighted in red. Overall, most uORFs seem to positively correlate with primary ORF TE, as previously reported for the human heart [2]. (**G**) Two rare examples of genes with strongly anti-correlating translation rates for the uORF vs. the primary ORF.

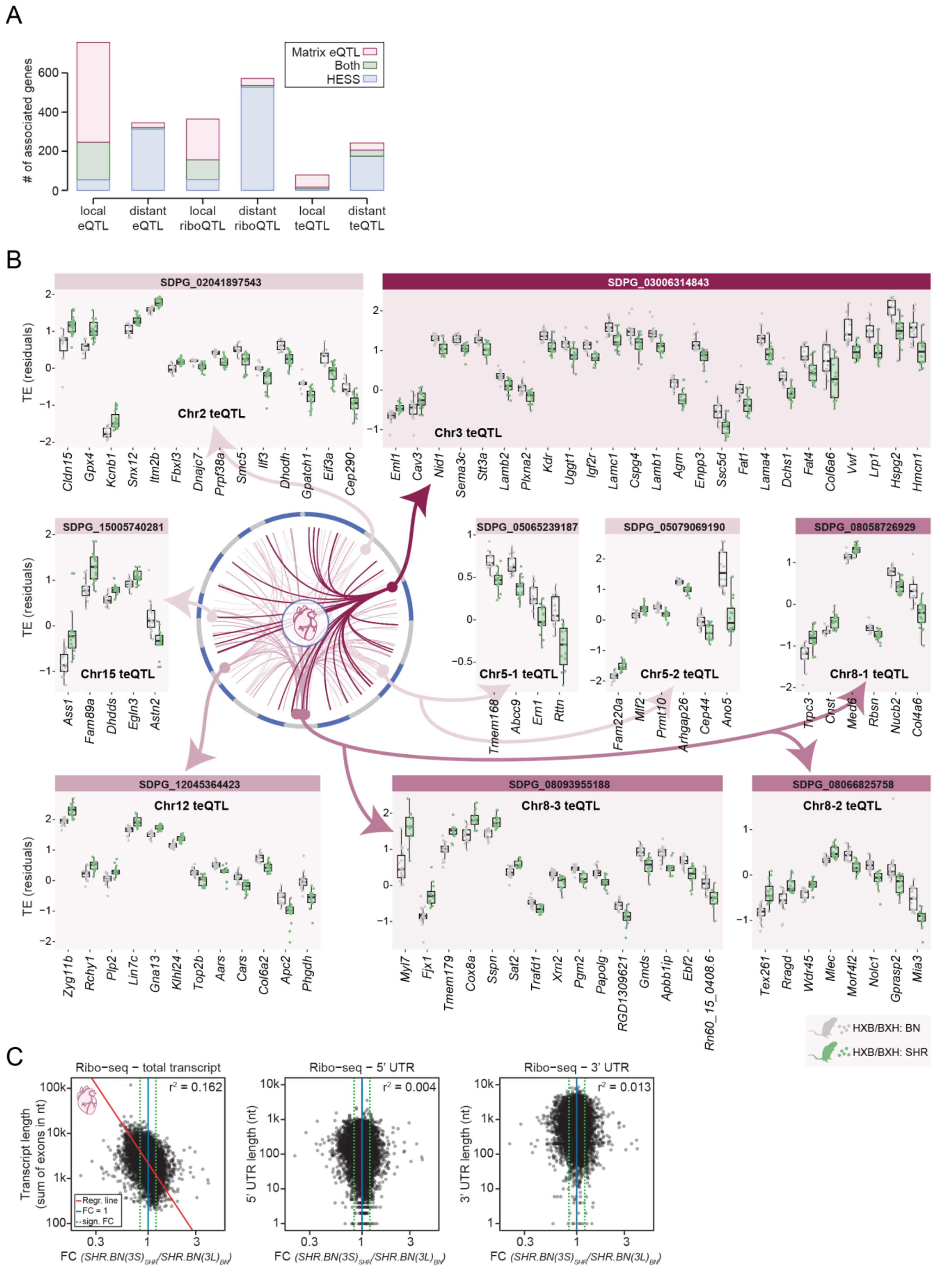

Additional File 1: Figure S3: The chromosome 3p teQTL regulates cardiac translation in a protein length-dependent manner, related to Figure 2. (**A**) Stacked bar plot showing the number of associated genes (i.e. with specific QTLs) in the rat heart, for Matrix eQTL, HESS or both. HESS is especially powerful for the detection of distant associations where a single locus can be linked to multiple genes. (**B**) Circos plot with all distant master regulatory teQTLs that associate with the TE of at least 5

genes (as in **Figure 2A**). For each identified distant QTL hotspot, gene-specific TE dot box plots are given for all associated genes. The teQTL coordinates, i.e. the genotype block linked to each set of genes, can be derived from the SDP ID (e.g. SDPG_03006314843 starts at Rat rn6 Chr3:6,314,843). Per teQTL, genes are ordered by effect size and directionality. See also **Additional File 5: Table S4**. (**C**) Scatter plots and square correlation coefficients (r^2) based on standardized major axis (SMA) values between total transcript length, 5' UTR length, or 3' UTR length versus the fold change (FC) in gene expression, as measured by Ribo-seq in congenic rat hearts. Obtained correlation coefficients (r^2) are lower than that of the comparison between CDS length and FC in translation (see **Figure 2E**), indicating that CDS length is the main determinant of the translational efficiency phenotype. For UTR length versus FC in gene expression, only cases with at least 10 DESeq2-normalized counts in both SHR.BN-(3L) and SHR.BN-(3S) rats are displayed. For total transcript length versus FC in gene expression, the correlation is significant (p-value < 2.2×10^{-16}; Test of correlation coefficient against zero) and the linear model based on fitted SMA method is displayed as a red line.

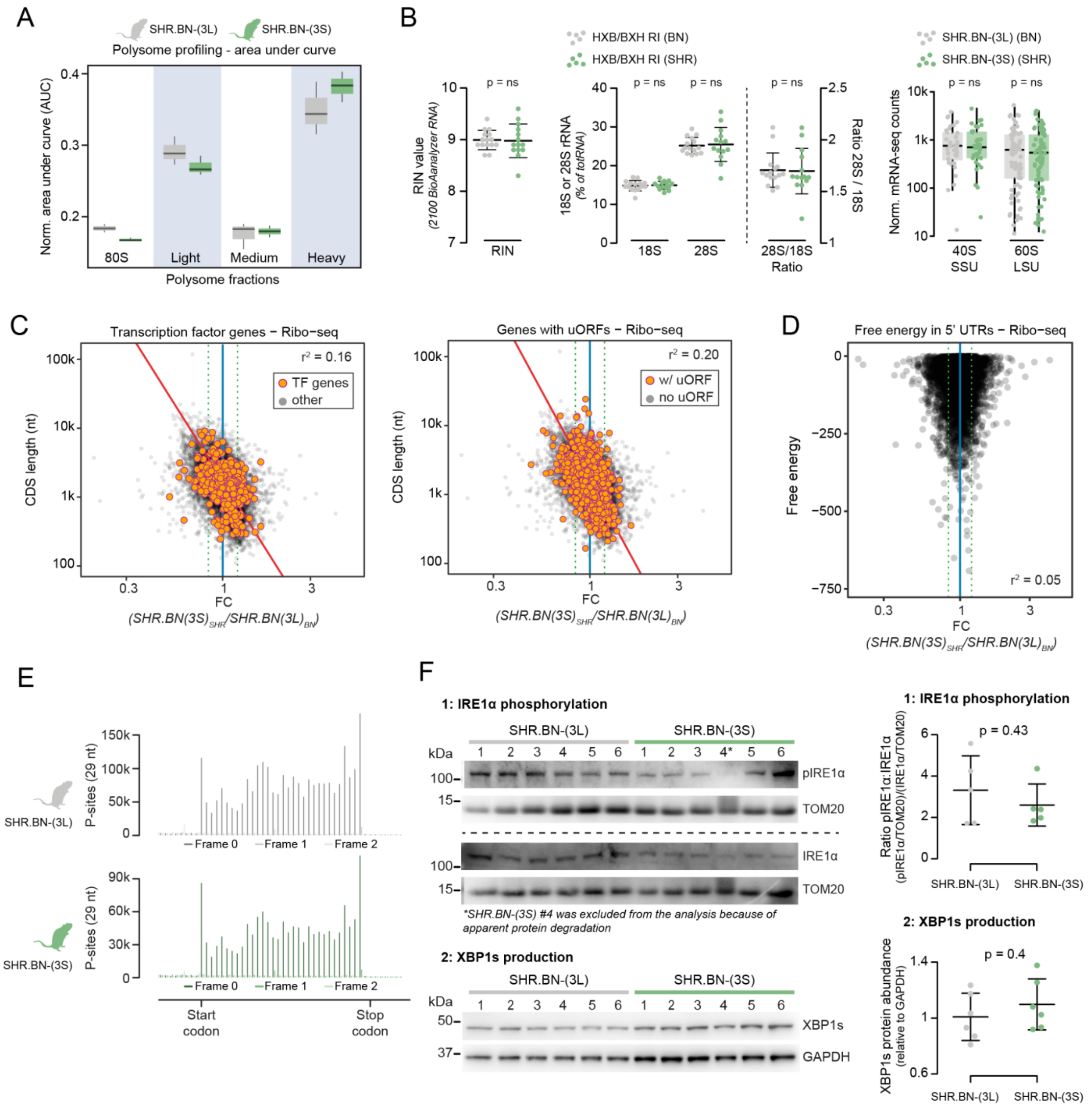

Additional File 1: Figure S4: Impaired ribosome assembly and half-mer formation drive the chromosome 3p teQTL, related to Figure 3. (**A**) Rat congenic line comparison of differences in polysomal configuration as measured by normalized area under curves (AUCs) of the polysome profiles, for SHR.BN-(3L) (grey) and SHR.BN-(3S) (green). (**B**) Left panel: dot plots with RIN values of HXB/BXH RI lines separated by Chr. 3p teQTL genotype. RIN values shown are calculated on the Agilent BioAnalyzer 2100 using the RNA nano assay. The middle panel contains dot plots of the HXB/BXH RI lines, with (i) 18S abundance, (ii) 28S abundance and (iii) 28S / 18S ratios, as calculated by the percentage of total RNA with an Agilent BioAnalyzer 2100 RNA nano assay. The right panel contains dot box plots comparing congenic rat line mean mRNA expression values for ribosomal protein genes involved in the structure of the 40S (SSU) and 60S (LSU) ribosomal subunits. Values are separated by local genotype at the chromosome 3p teQTL locus and only left ventricular heart tissue RNA data is shown. Error bars indicate mean values with standard deviation (SD). All these analyses indicate no imbalance between the production levels of both ribosomal subunits. (**C**) Scatter plot showing CDS length versus fold change (FC (SHR.BN-(3S) vs SHR.BN-(3L)) for Ribo-seq data, highlighting all genes with actively translated uORFs (left) and annotated as transcription factor (right) in the rat

heart. The square correlation coefficient (r^2) based on standardized major axis (SMA) is calculated using expression values of the subsets of genes only, and precisely matches that of the whole translatome (r^2 = 0.20), showing that there is no differential regulation of genes with uORFs or annotated as transcription factors as opposed to the full set of translated genes. **(D)** Scatter plot showing 5'UTR free energy length versus fold change (FC (SHR.BN-(3S) vs SHR.BN-(3L)) for Ribo-seq data. Free energies were calculated using RNAfold [3]. (**E**) Meta-gene codon 3-nt periodicity bar plots displaying P-sites of 29-nt ribosome footprints to illustrate the similarity in translation elongation rates between both congenic lines. The visualized data are a merger of all replicate SHR.BN-(3L) (grey, top) and SHR.BN-(3S) (green, bottom) Ribo-seq 29-nt footprints. Plots are generated with Ribo-seQC [4] and modified to only display the following sections of genes: (i) 25nt before and 33nt after the start codon, followed by (ii) 33nt from the middle of the CDS, and finally (iii) 33nt before and 25nt after the stop codon [4]. (**F**) Western blots and dot plots with quantification of band intensities for the ER stress markers IRE1-alha phosphorylation (normalized for IRE1-alpha and TOM20 expression; top) and XBP1s production (normalized for GAPDH expression; bottom). Error bars indicate mean values with SD. These protein level analyses show no difference in the expression or activation of typical ER stress response markers. Full size, uncropped blots with complete ladders are provided in **Additional File 6: Figure S6**.

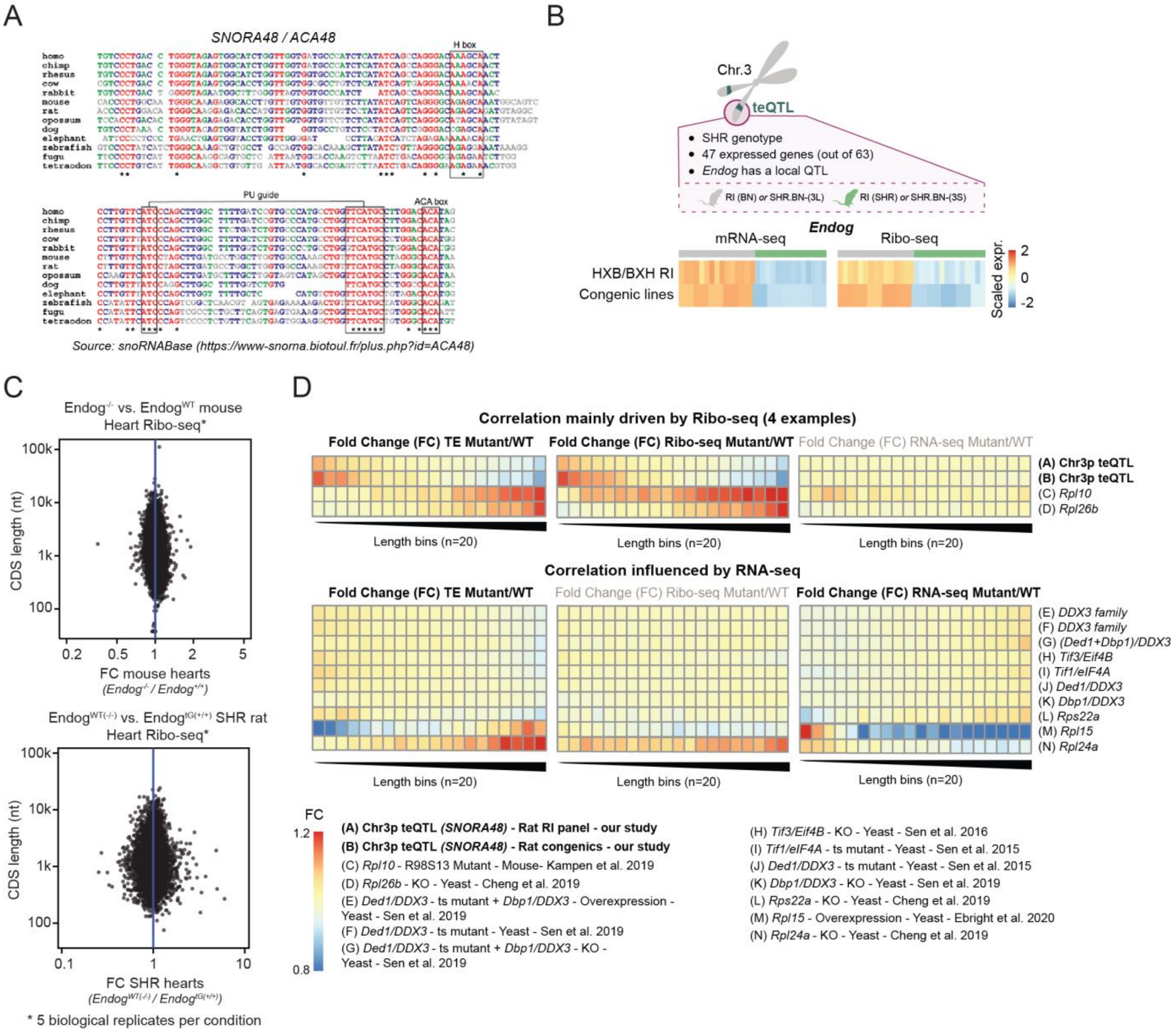

Additional File 1: Figure S5: Reduced translation initiation rates reinforce a pre-existing length-bias in TE. (**A**) Multi-species alignment for the H/ACA box snoRNA *SNORA48*, highlighting functional domains (H box, pseudouridylation guide, ACA box), as obtained from snoRNABase v3 (snoRNA-LBME-db; visited: March 2020) [5]. (**B**) Schematic visualization of the rat chromosome 3 teQTL and a summary of the expressed genes within this region. Expression values for *Endog* are given. Heatmaps show scaled and normalized expression values. (**C**) Scatter plots for CDS length versus the fold change (FC) in gene expression as measured by Ribo-seq in *Endog*$^{-/-}$ mice vs WT mice (left) and transgenic SHR/Ola rats with partially rescued Endog expression versus wild type SHR/Ola (right). Ribo-seq was performed on 5 hearts per condition (see also Additional File 2: Table S1), though reveals no correlation between CDS length and translation suggesting that *Endog* knockout or transgenic rescue alone is not sufficient to induce or ameliorate the CDS length-dependent shift in TE. (**D**) Heatmaps with CDS length versus fold change (FC Mutant vs Wild Type) for Ribo-seq, RNA-seq and translational efficiency data from a selection of studies of various translational machinery mutants. Absolute fold changes are given. Within each group, genes are divided into 20 equally-sized bins by increasing CDS length (left to right). Samples are sorted by Pearson's correlation coefficient (r, top to bottom), The top group comprises a selected set of 4 datasets where the correlation is mainly driven by Ribo-seq; see also Figure 4E. The bottom group comprises a set of 10 datasets where the TE correlation shift was (partly or completely) influenced by RNA-seq, which challenges the interpretation of the Ribo-seq data and suggests that absolute translation rates in wild type and mutant are equal and not influenced by CDS length (middle panel).

Uncropped western blots

1: IRE1a phosphorylation (phosphorylated IRE1a)

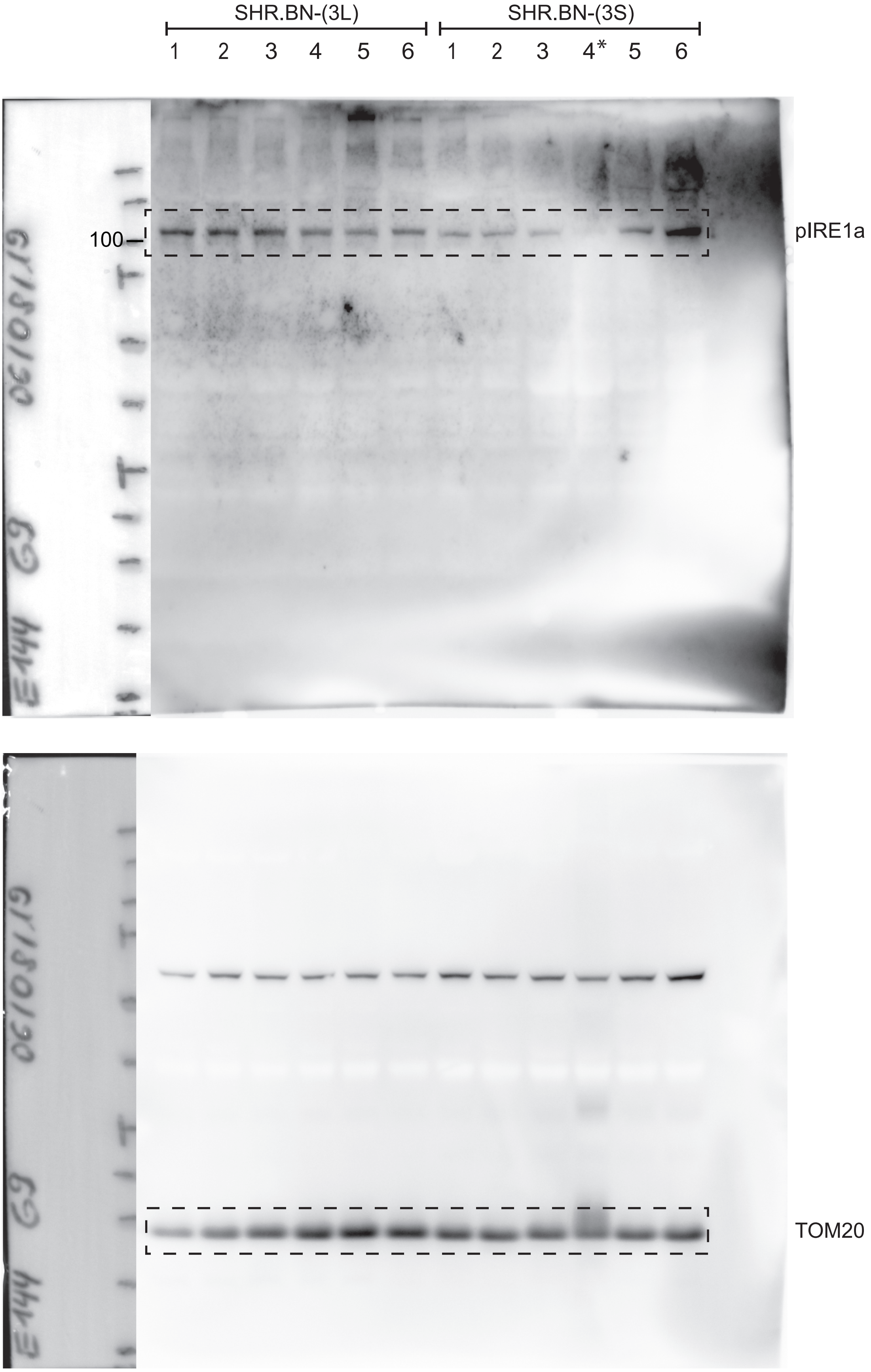

* excluded from analysis due to apparent protein degradation

Uncropped western blots

1: IRE1a phosphorylation (unphosphorylated IRE1a)

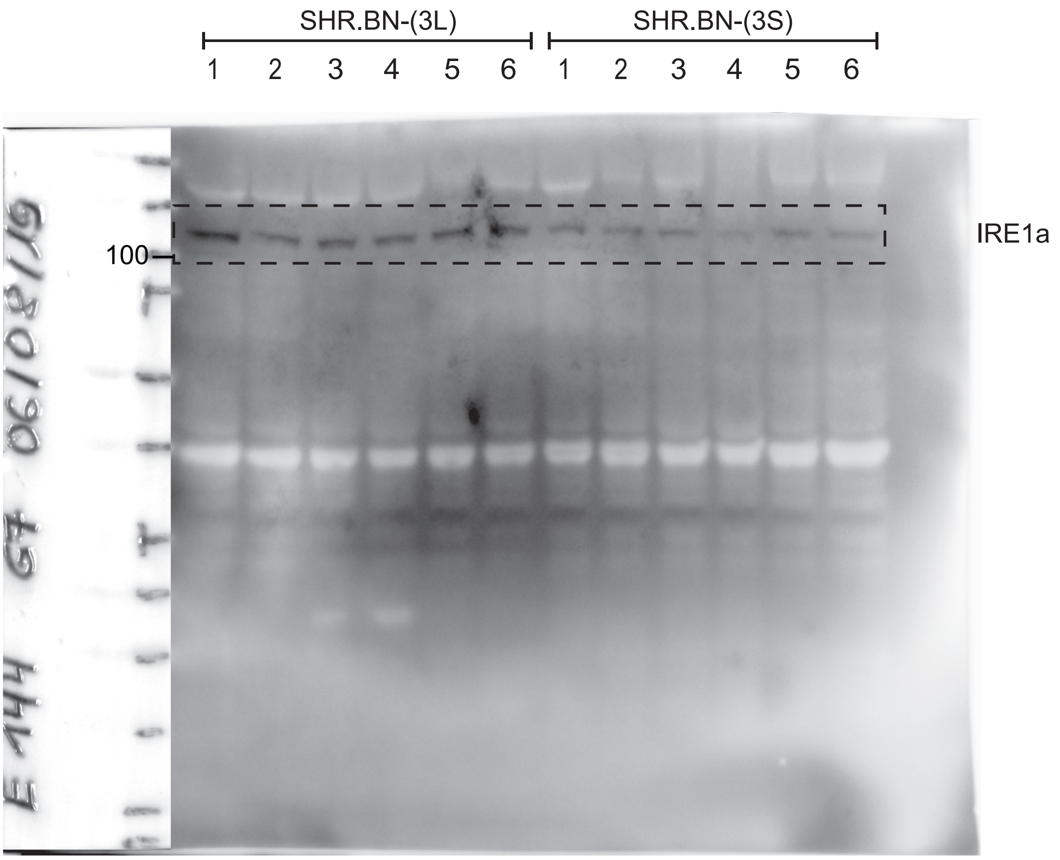

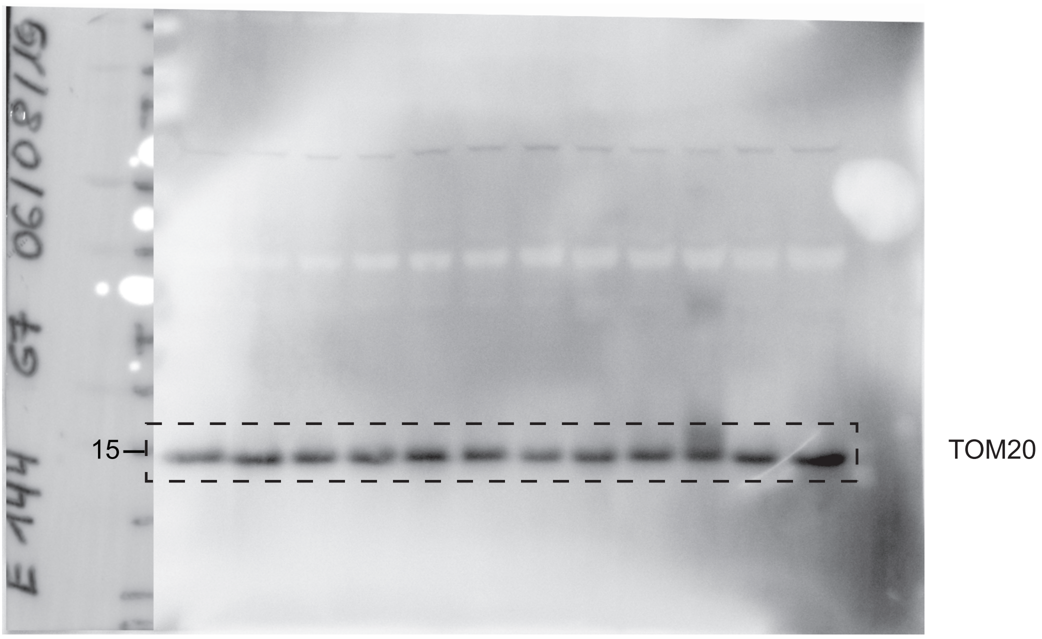

Uncropped western blots

2: XBP1s production

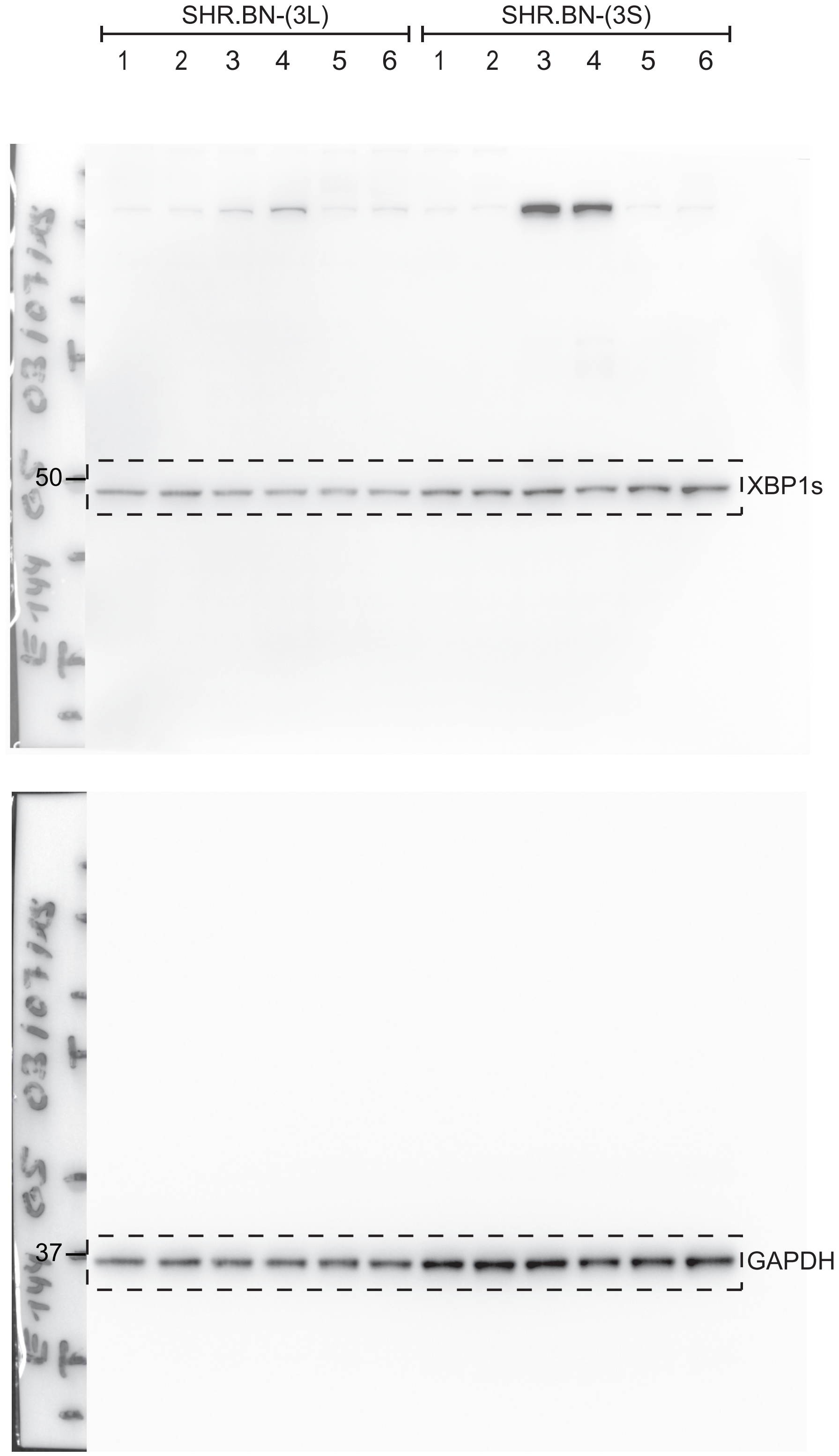

Additional File 1: Figure S6: This figure contains all uncropped Western blots and protein size markers for the experiments presented in Figure S4.

References

1. Cox J, Mann M. MaxQuant enables high peptide identification rates, individualized p.p.b.-range mass accuracies and proteome-wide protein quantification. Nat Biotechnol. 2008;

2. van Heesch S, Witte F, Schneider-Lunitz V, Schulz JF, Adami E, Faber AB, et al. The Translational Landscape of the Human Heart. Cell [Internet]. 2019;178:242-260.e29. Available from: https://linkinghub.elsevier.com/retrieve/pii/S0092867419305082

3. Lorenz R, Bernhart SH, Höner zu Siederdissen C, Tafer H, Flamm C, Stadler PF, et al. ViennaRNA Package 2.0. Algorithms Mol Biol [Internet]. BioMed Central; 2011 [cited 2020 Nov 19];6:26. Available from: https://almob.biomedcentral.com/articles/10.1186/1748-7188-6-26

4. Calviello L, Sydow D, Harnett D, Ohler U. Ribo-seQC: comprehensive analysis of cytoplasmic and organellar ribosome profiling data. bioRxiv. 2019;

5. Lestrade L, Weber MJ. snoRNA-LBME-db, a comprehensive database of human H/ACA and C/D box snoRNAs. Nucleic Acids Res [Internet]. 2006;34:D158-62. Available from: https://academic.oup.com/nar/article-lookup/doi/10.1093/nar/gkj002
