# Supplementary material for: A trans locus causes a ribosomopathy in hypertrophic hearts that affects mRNA translation in a protein length-dependent fashion
Source: Genome Biol. 2021 Jun 28;22:191. doi: 10.1186/s13059-021-02397-w (PMC8240307; doi:10.1186/s13059-021-02397-w)
Supplement: Supplementary file 7 — Additional file 7. Review history. [file 13059_2021_2397_MOESM7_ESM.docx]

Review History

**First round of review**

**Reviewer 1**

**Are you able to assess all statistics in the manuscript, including the appropriateness of statistical tests used?** Yes, and I have assessed the statistics in my report.

**Comments to author:**

The paper by and colleagues identify a locus and suggest to display an intergenic epistasis on cardiac hypertrophy phenotype. Two organs, liver and left-ventricular heart, of 30 recombinant in-bred rat lines, were sequenced (RNA-Seq and Ribo-Seq). Comparative analyses reveal a small set of genes translationally upregulated which mostly belong to the category ribosomal assembly. In a combined analysis of the RNA-Seq and Ribo-Seq (i.e. expressed as TE values) the authors see a shift in the TE values in a length-dependent manner, e.g. the TE values of short ORFs increase and those of long ORFs decrease. However, the effect seems subtle, the correlation coefficient is fairly low. The authors search for a magic regulator in the 5' UTRs (uORFs, Kozak - not to mention those are not the only regulatory elements) but do not find any.

Then the authors go on and use a sucrose-gradient fractionation of the ribosomes and quantify those profiles, which are largely established in the literature as qualitative. They perform RNA-seq of different fractions of those and use this largely qualitative analysis to calculate ribosomes and conclude that 'induces polysome half-mer formation'. [What does a half-mer mean in a context of the ribosome? Single subunit, but they have different size cannot be half? A single ribosome is called monosome, a half-mer in a polysome designating multiple ribosomes translating one message is even beyond any imagination what it could be]. Here, one starts wondering why the authors use a qualitative data to extract any numbers when they have quantitative data sets, Ribo-Seq combined with RNA-Seq, which give the position of translating ribosomes with nucleotide precision and the number of ribosomes per message, why using.

In the last part of the paper, the authors consider reanalyzing some published data sets, including such from ribosomal mutants lacking single ribosomal protein. By seeing few trends and limited correlation of some with their data, they conclude that down-regulation/knock-out of many translation factors (not only ribosomal proteins) affect efficiency of translation. Indeed, their reanalyses corroborate the conclusion of the single papers (from which the data originate), but do not reveal any insights to the major hypothesis in this paper.

Although the pathology mechanism of ribosomopathies and in particular the tissue specificity is still puzzling, the study fails to elucidate any insights into the problem. The problem stems from flows in the data interpretation, but also from lack of a clear concept and hypothesis, precluding the design of thoughtful experiments and analysis. A careful read of the review of Milis/Green (Science 2017; ref. 74 in the manuscript) which refer to the original concentration hypothesis of Lodish (Nature 1974) may help the authors designing better analysis of their data in addressing the observation of transcript-length specific translation alterations. Currently, the study is observatory, failing to reveal any mechanistic insights on the transcript-length specific translation and unfortunately provide no conceptual advance.

Few other comments:

1. The paper lacks any introduction and starts almost directly what the content and fining of the paper is. In general, the whole manuscript is written in a quite-difficult-to-read way. It started as ribosomopathy-focused paper claiming in the abstract to have find an epistasis locus which in the discussion turns out that it is a general modifier of translation. Neither is true, as the data do not support the connection to ribosomopathies and to translational regulation.

2. Through a constant intercalation of literature data/published evidence in their results section it is difficult to disentangle, what the authors really think they see in their data or what has been already seen and they just use it as interpretation of their data.

3. The used terminology is a bit off from what is established in the literature. An example, de novo initiation - such term does not exist. Initiation already designates that it is de novo. The authors should stay as scientific as possible in their writing and should avoid words more common for non-scientific outreach audience. Just to mention few one would wonder what they mean: 'changed ribosomal configurations', 'half-mer'.

4. The primary role of snoRNA48 is pseudouridylation on rRNA in the nucleus before subunit association, which in principle is a catalytic reaction. This makes me wonder why the authors expect an 'association with ribosome' and somehow design analysis to search for it. Failed pseudouridylation compromises the ribosomal assembly and hence decreases the concentration of the active ribosomes in the cytosol. Maybe this is the direction ot search for an effect of snoRNA48?

5. How an inbred line can be used to infer the human-to-human heterogeneity? (l. 79-80). Is it not more the heterogeneity of an outbreds?

**Reviewer 2**

**Are you able to assess all statistics in the manuscript, including the appropriateness of statistical tests used?** Yes, and I have assessed the statistics in my report.

**Comments to author:**

In this paper, Witte, Ruiz-Orera et al. used ribosome profiling in a rat recombinant inbred panel to map QTLs that affect translation efficiency. They discover that a locus known to affect cardiac hypertrophy also alters translation of multiple genes. Interestingly, this locus affects translation in a protein-length dependent manner. The authors validate this length-dependent translation effect in independently sampled animals, and show that translation gene mutants in humans and yeast also show varying degrees of length-dependent shifts in translational efficiency, leading the authors to propose a model under which pre-existing negative correlations between CDS length and translation are enhanced or reduced, driven by imbalance of translation initiation and re-initiation.

I really enjoyed this paper. Understanding genetic effects on the different layers of gene expression is important, in particular trans effects. Trans effects on translation have not been studied to date (to my knowledge), making this paper a timely and interesting contribution. The paper is extremely clearly written and beautifully presented. In particular, I applaud the authors for their exceptional figures. They are not only informative but also creative and attractive. The extensive figure legends add crucial additional information. Well done.

While the authors seem slightly disappointed that they weren't able to pinpoint the causal gene in the locus, I don't see this as a major weakness. Instead, the fact that Endog (a gene known to lead to heart phenotype) can be excluded as candidate gene for SNORA48 mis-regulation and the TE/CDS length phenotype is important information that will motivate additional research into this region.

Overall, this is a terrific paper. I only have one major and one minor comment:

Major:

Statistical power to detect QTLs is limited with 30 lines. Many false negatives are expected, which could account for the many kinds of apparently specific QTLs (e.g. uORF QTLs that were not also teQTLs, or tissue-specific QTLs). Indeed, the results shown in Figure 1F support this notion. At first glance, I thought that the first panel (for Casp4) was intended to show a QTL that was present in both tissues - visually, both tissues show higher TE in rats with the SHR genotype. The difference in significance between tissues could easily be due to a false negative in the liver. In fact, each of the three examples shows a difference in consistent direction, irrespective of significance. Two things should be done to address this:

1. Clearly acknowledge in the main text the limited statistical power that comes from using only 30 lines, and that this can result in false inferences of specificity.

2. For cases of discrepancies between QTL types (specifically, tissue specificity and ribo-QTLs that are not eQTLs), what fraction of QTLs show an effect in the same direction in the two data types? If, as a group, these QTLs really are specific, the direction of effect in the data in which no QTL was detected is expected to be random with respect to the direction of the detected QTL. By contrast, an excess of shared direction would point to more sharing of signal than can be detected using stringent QTL detection thresholds. These analyses should be added to the paper.

Minor:

Page 18 line 377: I suggest removing the word "Unfortunately".

**Reviewer 3**

**Are you able to assess all statistics in the manuscript, including the appropriateness of statistical tests used?** Yes, and I have assessed the statistics in my report.

**Comments to author:**

In this manuscript, Witte et al. performed genotyping, RNA-seq and Ribo-seq in 30 rat recombinant inbred lines. Using this framework, they identify several eQTLs, ribo-QTLs and translational-efficiency QTLs (teQTLs). They detect a region in chromosome 3 that seems to be associated with the transcriptional efficiency of multiple genes in trans. They then confirm that finding using an engineered rat line carrying only the mutant segment compared to an isogenic control and proceed with some further characterization to show that the identified genetic variation in chromosome 3 induces a ribosomopathy leading to polysome half-mers. There are a few methodological concerns that need to be addressed:

1. The authors need to provide more details on how the recombinant inbred rat lines were generated and selected. What is the degree of relatedness between the different lines and how could that affect or confound results in QTL calling?

2. Similar to the above, it is unclear whether the authors accounted for population stratification and relatedness in their QTL analyses. The authors should present analyses attempting to correct for those common confounders.

3. Also accounting for cryptic confounding using either PEER or SVA applied to the molecular data would be advised in such datasets. The number of identified QTLs seem out of proportion to the number of test subjects which could be partially accounted for by the decrease in multiple testing burden by aggregating variants in SDAs but could also suggest inflation from uncontrolled confounding.

4. In addition, read mapping errors due to cross-mappability could explain some/all of the detected trans-QTLs. The authors should check for cross-mappability of their trans-eGenes with any cis-genes and present read-pileups for cross-mappable genes stratified by genotype of the corresponding trans SDA to reassure that is not the case. [PMID: 30613398]

5. Do the QTLs identified in this dataset replicate in other rat QTL datasets? That would provide some more confidence in the findings

6. Allowing multimapping regions in detecting snoRNAs is ill-advised as it further raises concern for the accuracy of QTL detection for those transcripts.

7. This is especially relevant for SNORA48 that the authors propose as the mechanism for the observed ribosomopathy in the chromosome 3 locus. The case for it being the cause of the ribosomopathy seems weak with the only supporting data being its decreased ribosome association in mutant hearts. Does overexpression of the target RNA in SHR-BN-(3S) rats rescue the observed ribosomopathy?

8. Minor comment: Co-localization has evolved to have a specific meaning implying a shared causal signal between two different datasets. The authors use it to mean positional overlap. Would suggest rephrasing throughout.

**Authors Response**

**Point-by-point responses to the reviewers’ comments:**

**Reviewer #1**: The paper by and colleagues identify a locus and suggest to display an intergenic epistasis on cardiac hypertrophy phenotype. Two organs, liver and left-ventricular heart, of 30 recombinant in-bred rat lines, were sequenced (RNA-Seq and Ribo-Seq). Comparative analyses reveal a small set of genes translationally upregulated which mostly belong to the category ribosomal assembly.

The description provided by Reviewer 1 does not accurately reflect our study: none of the presented QTL mapping analyses (either the *cis* or *trans* results) focus on a set of ribosome assembly genes, and in no part of our work we touch on the concept of epistasis. We distinguish between local and distant genetic influences on translational control, and put emphasis on the novel observation that large parts of translational control are orchestrated in a tissue specific manner, frequently through distant loci, reflecting *trans*-acting mechanisms. We identify multiple distant genomic hotspots using two independent methodologies (matrixEQTL (*Shabalin et al., Bioinformatics 2012*) and HESS (*Lewin et al., Bioinformatics 2016*) that each harbor master regulators of translation. We continue to mechanistically dissect one of these loci. This is the first illustration of distant translational efficiency QTL mapping in mammals, the first instance where local or distant QTLs are investigated in a complex disease setting, and the first time a distant locus is shown to trigger a global translational machinery deficiency that ultimately causes heart failure.

In a combined analysis of the RNA-Seq and Ribo-Seq (i.e. expressed as TE values) the authors see a shift in the TE values in a length-dependent manner, e.g. the TE values of short ORFs increase and those of long ORFs decrease. However, the effect seems subtle, the correlation coefficient is fairly low.

The CDS length-specific correlation effect is highly genome-wide significant (with a p-value < 2.2 x 10^-16^), absent in liver, and independently reproducible in each comparison when separated by genotype (see the figure below for a 3 vs 3 comparison). The length-specific correlation effect is even higher for sarcomere proteins that compose the basic unit of muscle fiber (r^2^=0.60, p-value < 2.2 x 10^-16^; Figure 3G, p15). Overall, the translatome-wide length effect is among the strongest observed for any ribosome deficiency (as outlined in figure 4E, p22, after comparing the effect with 9 published datasets in human, mouse, and yeast), and is also clearly visible in the independently performed polysome profiling experiments, which per definition makes this not a subtle effect. On the individual gene level (e.g., Figure 2F, p11), the shift in translation is highly reproducible and significant, even after genome-wide correction and extensive permutation testing.

**Reproducibility of the CDS-length correlation effect across comparisons separated by genotype.** Scatter plots, square correlation coefficients (r^2^), and correlation p-values (P) based on standardized major axis (SMA) values between coding sequence (CDS) length and the fold change (FC) in gene expression, as measured by Ribo-seq in a 3 vs 3 comparisons of congenic rat hearts. The FC in translation reproduces the global length effect observed for the Chr. 3p teQTL identified in the HXB/BXH RI panel. The linear model based on fitted SMA method is displayed as a green line.

The authors search for a magic regulator in the 5' UTRs (uORFs, Kozak - not to mention those are not the only regulatory elements) but do not find any.

It seems that the Reviewer mis-interpreted the context in which uORFs were investigated. Genetic effects on Kozak sequences, local translation initiation context and uORF translation were solely investigated in order to find a mechanistic explanation for local translational efficiency QTLs (Figure S1 and results in Section *“Local teQTLs are mechanistically independent of upstream ORFs”*; p7-8). As the aforementioned elements are key local regulators of translation, it is rational to evaluate whether genetic effects that impact these elements explain the *cis* mechanisms of action for local teQTLs. In addition, work by Cenik and colleagues (Genome Res 2015, PMID: [26297486](https://www.ncbi.nlm.nih.gov/pubmed/26297486)) points to genetic variants presumably affecting translation initiation context and uORFs as main potential mechanisms for local teQTLs. As our system provides more power to investigate this *cis* relationship, we felt it necessary to report that we could not validate a (quantitative) role for uORFs in the etiology of local teQTLs, nor that we found any genetic mutations impacting translation initiation in *cis*. Importantly, none of these local QTL analyses have been performed in relation to the distant QTL results (incl. the protein-coding length dependent shift in TE), though this seems to be wrongly perceived by the Reviewer. Also, we clearly stated in our manuscript that other additional regulatory effects in UTRs could regulate translation, such as RNA folding structures, methylation sites, or RNA binding protein motifs (p9, lines 177-179).

Then the authors go on and use a sucrose-gradient fractionation of the ribosomes and quantify those profiles, which are largely established in the literature as qualitative. They perform RNA-seq of different fractions of those and use this largely qualitative analysis to calculate ribosomes and conclude that 'induces polysome half-mer formation'. Here, one starts wondering why the authors use a qualitative data to extract any numbers when they have quantitative data sets, Ribo-Seq combined with RNA-Seq, which give the position of translating ribosomes with nucleotide precision and the number of ribosomes per message, why using.

The polysome fractionation experiments serve as a means to compare ribosomal distributions (the number of ribosomes the mRNA is associated with) as a measure of translational activity. This sheds light on the mechanistic basis of any potential translational deficiency, in this case clearly demonstrating that the translational status is severely hampered in diseased hearts. The use of RNA-seq derived from the four isolated fractions confirms and adds additional evidence for the observed length-specific effect that was revealed by using Ribo-seq. The polysome data also serve as a technology-independent control for the Ribo-seq data: the procedures between both techniques are different, excluding procedure-specific effects (i.e. polysome profiling does not depend on a nuclease treatment, sequencing reads are longer and paired-end, entire transcripts and not footprints are isolated, and physical association with ribosomes is visually and experimentally controlled for). The unique advantages and limitations of both Ribo-seq and polysome profiling have frequently been outlined, amongst others by Nicholas Ingolia - the developer of the Ribo-seq procedure (e.g. *Ingolia et al., Nat Prot 2012; Nat Rev Genet 2014*).

We have extensive experience with the quantitative and qualitative analysis of both Ribo-seq and Polysome profiling data (e.g. *van Heesch et al., Genome Biol 2014; Schafer et al., Nat Comm 2015; van Heesch et al., Cell 2019; Gaertner & van Heesch et al., eLife 2020*), and have, similar to others in the field (e.g. *Bunnik et al., Genome Biol 2013; Sterne-Weiler et al., Genome Res 2013; Kronja et al., Cell Rep 2014; Floor & Doudna, eLife 2016; Blair et al., Cell Rep 2017*; *Chassé et al., NAR 2017;* *Tiemy Pereira et al., BMC Genomics 2019*), analyzed polysome profiling RNA-seq data in a quantitative way. Even prior to the widespread use of RNA-seq, it was common practice to combine polysome profiling with expression microarrays for the quantitative analysis of translational control (e.g. *Arava et al., PNAS 2003* and reviewed in: *Larsson et al., CSH Persp Biol 2013* and *King & Gerber, Brief Funct Genomics 2014*).

The polysome profiles also served a qualitative purpose for our study, as they pointed us to a ribosome assembly and initiation defect, characterized by polysome half-mers and the mRNA-specific length-dependent shifts in the number of associated ribosomes (Figure 3, p15).

[What does a half-mer mean in a context of the ribosome? Single subunit, but they have different size cannot be half? A single ribosome is called monosome, a half-mer in a polysome designating multiple ribosomes translating one message is even beyond any imagination what it could be].

Half-mers are a very common phenotype in polysome profiles, which indicate a translation initiation deficiency. The term polysome half-mer (or halfmer) describes the imperfect assembly of the preinitiation complex on the mRNA, which is not instantly matched by a 60S subunit to form a functional 80S monosome (e.g., *Kappen et al., Biochemistry 1976; Helser et al., MCB 1981; Rotenberg et al., Genes & Dev 1988*). Any significant delay in subunit joining reflects an impairment in translation initiation, which becomes visible in polysome profiles as an additional ‘shoulder’ to each additional ribosome peak (indicated by vertical arrows in Figure 3C, p15). For instance, an mRNA bound by a single 80S monosome and a subsequent 43S or 48S pre-initiation complex (PIC) waiting for a 60S subunit to join, would migrate at a different size in our sucrose gradient than a single 80S monosome alone. Or, as Rotenberg (1988) described this phenomenon: *“Most strikingly, there are discrete peaks sedimenting at positions in the gradient intermediate to those polyribosome peaks containing integral numbers of ribosomes“*.

We believe we have very clearly introduced and covered the concept of polysome half-mers in the text (p13, lines 246-254), but have added additional references (the added references are highlighted in bold in the paraphrased text below) to the revised version of the text, referring to more papers where half-mers have been described previously:

*“Polysome profiles of SHR.BN-(3S) rats showed heavily altered ribosomal configurations compared to SHR.BN-(3L) (Figure 3A+B and Figure S3A), exemplified by “shoulders” accompanying each polysome peak indicative of polysome half-mer formation (Figure 3C)* ***(Rotenberg et al., Genes & Dev 1988; Li et al., PLOS Biol 2009).*** *Polysome half-mers are formed when the 43S preinitiation complex does not instantly join the large 60S ribosomal subunit to form a functional 80S monosome. This stalls translation initiation - the rate-limiting step of RNA translation and therefore a main determinant of TE [28,43,44]. Half-mers arise because of ribosome biogenesis defects, caused by the underproduction of 60S subunits [45] or impaired subunit joining [46,47].”*

What makes proper understanding of translation initiation and half-mer formation particularly important for our work and proposed mechanism, is that half-mers are frequently observed upon deletion of factors crucial for ribosome biogenesis and the establishment of pre-initiation complexes (e.g. see a systematic interrogation of biogenesis factors in *Li et al., PLOS Biol 2009*). This includes snoRNAs such as *U24* / *SNORD24* (*Kouba et al., NAR 2012; Thompson et al., eLife 2016*), which also drives a length-dependent shift in translation upon KO (*see Figure 4D for a comparison of SNORD24-/- with our data*).

For further reading we would like to refer the Reviewer to e.g. *Eisinger et al., MCB 1997; King et al., Mol Cell 2003; Nielsen et al., EMBO J 2004; Colón-Ramos et al., NSMB 2006; Meyer et al., PNAS 2007* and *Li et al., PLOS Biol 2009*.

In the last part of the paper, the authors consider reanalyzing some published data sets, including such from ribosomal mutants lacking single ribosomal protein. By seeing few trends and limited correlation of some with their data, they conclude that down-regulation/knock-out of many translation factors (not only ribosomal proteins) affect efficiency of translation. Indeed, their reanalyses corroborate the conclusion of the single papers (from which the data originate), but do not reveal any insights to the major hypothesis in this paper.

We are not corroborating the conclusions of the selected articles where the analyzed data comes from, as only few of these studies reported a CDS length-specific effect on translational regulation. Instead, we introduce this meta-analysis as additional proof to confirm our hypothesis and show that the presented length-specific effect is in line with other ribosomopathies, being a common hallmark of many of these disorders. We show that this trend can go in both directions, resulting either in the (relative) translational downregulation of long CDSs and upregulation of short CDSs, or vice versa. We mechanistically connect these observations to imbalances in the efficiencies of (*de novo*) initiation versus reinitiation after ribosome recycling - both of which contribute to the translational output of a single gene.

In addition, and as we mentioned above, the comparison of the ribosome biogenesis defect with other ribosomopathies reveals that our reported length effect is not subtle, and it is among the strongest observed for any ribosome deficiency. Most importantly, our data are *in vivo* and come from heart tissue within a complex disease context (whereas most of the other datasets are from yeast or *in vitro* mammalian cell culture experiments). We have included (to our knowledge) all published mutant Ribo-seq datasets that affect ribosome assembly, translation initiation, or the ribosome itself. We believe this is a valid, appropriately introduced, and above all very important reanalysis of (in part) previously published datasets.

Although the pathology mechanism of ribosomopathies and in particular the tissue specificity is still puzzling, the study fails to elucidate any insights into the problem. The problem stems from flows in the data interpretation, but also from lack of a clear concept and hypothesis, precluding the design of thoughtful experiments and analysis. A careful read of the review of Milis/Green (Science 2017; ref. 74 in the manuscript) which refer to the original concentration hypothesis of Lodish (Nature 1974) may help the authors designing better analysis of their data in addressing the observation of transcript-length specific translation alterations. Currently, the study is observatory, failing to reveal any mechanistic insights on the transcript-length specific translation and unfortunately provide no conceptual advance.

We strongly disagree with this assessment. We are very familiar with the Mills & Green review on ribosomopathies (*Science 2017*). In fact, the ribosome concentration hypothesis (*Lodish, Nature 1974*) is not at all contrasting our work and we have clarified this below and in the revised version of our manuscript. Similar to our working hypothesis, the presented kinetic rate equation model acknowledges a crucial role for the RNA-specific rate of translation initiation (ki). Or, as Lodish (*Nature 1974*) wrote: *“A principal result of these calculations is that any reduction in the rates of polypeptide chain initiation steps at or before binding of mRNA will result in preferential inhibition of translation of mRNAs with lower initiation rate constants (the poorer mRNAs).”*

Q = protein synthesis rate

m = mRNA expression level

R = ribosome concentration

ki = initiation rate

ke = termination rate (set to 1.0)

L = footprint length (10 codons)

What exactly defines differences in initiation rates remains uncertain from the discussed literature, but is put forward by Mills & Green (*Science 2017*) to likely depend on the class of mRNAs (e.g., transcription factors) and on local *cis*-acting elements that influence translation (e.g., uORFs, highly structured UTRs, IRESs, multiple upstream start sites):

*“The translation of certain mRNAs with low initiation rates ki, such as those with highly structured 5′UTRs, uORFs, IRES elements, multiple upstream start sites, or poor initiation contexts, will likely be impaired by reduced ribosome availability.” [...] “Conversely, this same analysis reveals that mRNAs with low initiation rates (e.g., those encoding hormones, transcription factors, certain Hox proteins, and GATA1) will be poor substrates for translation when ribosome availability is limited. Thus, the sensitivity of specific cells to RP mutations could result from selective reduction in the translation of particular mRNAs with inherently low translational efficiencies.”*

In contrast to these suggestions, we find that the most drastic changes in initiation rates are primarily determined by coding sequence (CDS) length, and not by length of the complete mRNA, or by factors that generally result in modest initiation such as 5’ UTR length (Figure S2C, p60) or the presence of uORFs (Figure S3E, p62). In fact, genes with uORFs are equally likely to be translational up- or downregulated (Figure S3E, p62) and calculations of the impact of 5’ UTR structure (as measured by free energy) on the fold change in translational efficiency (FC in TE; x-axis) have no influence on TE in the diseased rat hearts (see figure below; left panel - *figure added as Figure S3F in the revised version of the manuscript, p62*). Similarly, the proposed functional groups of mRNAs with inherently low initiation rates, such as transcription factors, display no (additional) changes in TE that can be attributed to such an inherently low initiation constant - the main determinant stays CDS length (see figure below; right panel - *figure added as Figure S3E to the revised version of the manuscript, p62*).

**Effect of 5’ UTR free energies and transcription factors in the CDS-length correlation effect. (left panel)** Scatter plot showing 5’UTR free energy length versus fold change (FC (SHR.BN-(3S) vs SHR.BN-(3L)) for Ribo-seq data. (**right panel**) Scatter plot showing CDS length versus fold change (FC (SHR.BN-(3S) vs SHR.BN-(3L)) for Ribo-seq data, highlighting all genes annotated as transcription factor in the rat heart. The square correlation coefficient (r^2^) based on standardized major axis (SMA) is calculated using expression values of this subset of genes only.

It is very likely, and in fact supported by the presence of half-mers in our polysome profiles, that the (absolute) availability of fully assembled 80S ribosomes is reduced in the diseased hearts. However, upon such reduced functional ribosome availability, we simply find no evidence for the proposed specific subsets of mRNAs with inherently lower initiation rates (examples given above) to behave any differently from all other translated mRNAs. This is further supported by the absence of any difference in ribosome footprint distribution or elongation rates across the mRNAs (Figure S2C, p60) and the length-dependent redistribution of the number of actively translating ribosomes per mRNA (Figure 3F, p15).

For these reasons, we put forward the initiation-reinitiation model presented by Rogers et al. (*PLOS Comp Bio 2017*) as the primary determinant of mRNA-specific initiation rates. According to this model, differences in the frequency of translation reinitiation are responsible for length-dependent changes in TE, which can explain a length-dependent shift in TE when either initiation or reinitiation efficiencies are affected. Reinitiation frequencies depend directly on the length of the translated coding sequence (a shorter sequence is translated faster), making a previously recruited ribosome readily available for the next round of translation. Deviations in initiation, but not reinitiation rates (and vice versa) are accurately modeled by Rogers et al. and perfectly match the CDS length-dependent shifts in TE that we observe in ribosome mutant datasets of ourselves and others, as presented in Figure 4E, p22. Upon reduced initiation rates, mRNAs with shorter CDSs (and thus inherently higher frequencies of reinitiation) are least affected, whereas mRNAs with long CDSs show marked reductions in TE. All of this appears (largely) independent of other canonical *cis* modulators of translation, as discussed above.

Few other comments:

1. The paper lacks any introduction and starts almost directly what the content and fining of the paper is. In general, the whole manuscript is written in a quite-difficult-to-read way. It started as ribosomopathy-focused paper claiming in the abstract to have find an epistasis locus which in the discussion turns out that it is a general modifier of translation. Neither is true, as the data do not support the connection to ribosomopathies and to translational regulation.

We respectfully disagree - we have infused the general background with a gradual introduction to our work, providing the relevant topics with the appropriate background where necessary. As corroborated (and even applauded upon) by Reviewer 2, we believe our manuscript is clearly written and graphically illustrated, with the right level of detail, even for the broad readership of Genome Biology. Again, this paper does not investigate epistasis and it appears that the Reviewer does not appreciate that the translational phenotype is caused by a genetic defect. The genetic locus is the cause of the ‘general modifier of translation’. We have more clearly emphasized this in the revised manuscript abstract, also to better emphasize the interdisciplinarity of our work.

2. Through a constant intercalation of literature data/published evidence in their results section it is difficult to disentangle, what the authors really think they see in their data or what has been already seen and they just use it as interpretation of their data.

All presented data is specifically generated for this study, except for the reanalyzed ribosomal mutant data in Figure 4E, p22. Where necessary, in both the results and discussion section, we reflect on previous literature to put our results and observations in perspective. We understand that our manuscript is information-dense and that it spans multiple fields in an interdisciplinary way (genetics research, systems biology, translational regulation and ribosome biology). However, we believe our results are clearly presented - both textually and visually - and this seems to be appreciated by, or not be problematic for, Reviewers 2 and 3.

3. The used terminology is a bit off from what is established in the literature. An example, de novo initiation - such term does not exist. Initiation already designates that it is de novo. The authors should stay as scientific as possible in their writing and should avoid words more common for non-scientific outreach audience. Just to mention few one would wonder what they mean: 'changed ribosomal configurations', 'half-mer'.

We adapted the term ‘*de novo* initiation’ from previous literature (e.g. *Kopeina et al., NAR 2008; Osterman et al., NAR 2013; Marshall et al., J R Soc Interface 2014; Jagannathan et al., RNA 2014; Rogers et al., PLOS Comp Biol 2017; Huber et al., Nat Comms 2019*) to help the reader distinguish the new recruitment and assembly of ribosomal subunits from the pool of available cytosolic ribosomes, versus the reinitiation of locally available, recently terminated ribosomes (through recycling at the same mRNA). However, we would be happy to remove '*de novo*', if the distinction between initiation and reinitiation is evident enough for the Reviewer, Editor, and readership of Genome Biology.

We have used the term ‘changed ribosomal configurations’ to indicate a shift in the mono- and polysomal occupancy of mRNAs, which became apparent from the polysome profiling experiments. We understand that the term ‘configuration’ can be confused with a conformational change of an individual ribosome and have adjusted this to ‘changed mono- and polyribosomal occupancy’ of mRNAs in the revised version of the manuscript.

For the use of the term ‘half-mer’ please see our previous explanation above.

4. The primary role of snoRNA48 is pseudouridylation on rRNA in the nucleus before subunit association, which in principle is a catalytic reaction. This makes me wonder why the authors expect an 'association with ribosome' and somehow design analysis to search for it. Failed pseudouridylation compromises the ribosomal assembly and hence decreases the concentration of the active ribosomes in the cytosol. Maybe this is the direction ot search for an effect of snoRNA48?

The differential presence of *SNORA48* became evident from the Ribo-seq data and was absent in total RNA-seq data (where snoRNAs were also captured; Figure 4C-D). We show that, while overall *SNORA48* production levels remain constant, cytoplasmic (Ribo-seq captured) *SNORA48* is strongly reduced in diseased hearts. Our data provide evidence for *SNORA48* being the highest expressed snoRNA in rat hearts, while interestingly being the only differential snoRNA in diseased hearts - in both the HXB/BXH RI panel, as well as the congenic lines. The distant genetic association between *SNORA48* abundance and the teQTL is highly significant (i.e. the association with translational regulation).

Although snoRNAs can associate with ribosomes when effectively exported into the cytoplasm during stress conditions (*Mleczko et al., Scientific Reports 2019*), the snoRNA signal in our Ribo-seq data may also come from alternative RNP complexes captured within the footprinting procedure (*Ji et al., Nat Biotech 2016*). Because we have not demonstrated a physical association of *SNORA48* with cytosolic ribosomes, we have clarified this in the revised version of our manuscript.

We would like to point out that the precise role of *SNORA48* in ribosome biogenesis still needs to be established. Based on sequence complementarity, *SNORA48* has been predicted to pseudouridylate specific residues of 28S rRNA. However, its precise function, target sites and mechanism of action have not been investigated or validated experimentally. We believe our observations warrant follow-up studies on the precise role of *SNORA48* in cardiac health and disease.

5. How an inbred line can be used to infer the human-to-human heterogeneity? (l. 79-80). Is it not more the heterogeneity of an outbreds?

The two parental lines that founded the RI panel share an extent of genetic variability, i.e., a number of single nucleotide variants, that is very comparable to that of two unrelated individuals (± 3M positions). Recombinant inbred lines can be seen as genetic mosaics of these two parental lines, which each have been made fully inbred again through repeated brother-sister mating (as described in the introduction, methods, results and shown in Figure 1, p9). This makes for a renewable and very well controlled experimental genetic system for which each of the two genotypes at any given locus is replicated by, on average, 15 animals. As the parental lines harbor complex disease phenotypes that are often polygenic (e.g., metabolic syndrome, hypertension, blood pressure independent hypertrophy, etc), the genetic basis of these traits can be precisely mapped within this panel. This gives this system the unique advantage of being suitable for molecular and physiological QTL analyses, with the added benefit that it provides sufficient power for the detection of distant associations in a very controlled way - this is unique for genetic studies in mammalian model systems.

We agree with the Reviewer that no model system can fully recapitulate human genetic heterogeneity and we have toned down (l 79-80) respectively. The number of translatome datasets presented in this study (94 Ribo-seq datasets (RI, transgenic, congenic and KO animals) and 24 polysome profiling RNA-seq datasets) is already the largest translatome effort published to date - for *any* species.

**Reviewer #2:** In this paper, Witte, Ruiz-Orera et al. used ribosome profiling in a rat recombinant inbred panel to map QTLs that affect translation efficiency. They discover that a locus known to affect cardiac hypertrophy also alters translation of multiple genes. Interestingly, this locus affects translation in a protein-length dependent manner. The authors validate this length-dependent translation effect in independently sampled animals, and show that translation gene mutants in humans and yeast also show varying degrees of length-dependent shifts in translational efficiency, leading the authors to propose a model under which pre-existing negative correlations between CDS length and translation are enhanced or reduced, driven by imbalance of translation initiation and re-initiation.

I really enjoyed this paper. Understanding genetic effects on the different layers of gene expression is important, in particular trans effects. Trans effects on translation have not been studied to date (to my knowledge), making this paper a timely and interesting contribution. The paper is extremely clearly written and beautifully presented. In particular, I applaud the authors for their exceptional figures. They are not only informative but also creative and attractive. The extensive figure legends add crucial additional information. Well done.

While the authors seem slightly disappointed that they weren't able to pinpoint the causal gene in the locus, I don't see this as a major weakness. Instead, the fact that Endog (a gene known to lead to heart phenotype) can be excluded as candidate gene for SNORA48 mis-regulation and the TE/CDS length phenotype is important information that will motivate additional research into this region.

Overall, this is a terrific paper. I only have one major and one minor comment:

Major:

Statistical power to detect QTLs is limited with 30 lines. Many false negatives are expected, which could account for the many kinds of apparently specific QTLs (e.g. uORF QTLs that were not also teQTLs, or tissue-specific QTLs). Indeed, the results shown in Figure 1F support this notion. At first glance, I thought that the first panel (for Casp4) was intended to show a QTL that was present in both tissues - visually, both tissues show higher TE in rats with the SHR genotype. The difference in significance between tissues could easily be due to a false negative in the liver. In fact, each of the three examples shows a difference in consistent direction, irrespective of significance. Two things should be done to address this:

1. Clearly acknowledge in the main text the limited statistical power that comes from using only 30 lines, and that this can result in false inferences of specificity.

First of all, we would like to thank the Reviewer for her/his appreciation of our work. It is indeed true that QTLs with low effect sizes are more likely to be missed than they would be in populations of larger sizes (see power calculation below). We have now better acknowledged this in the revised manuscript, accompanied by the at point 2 suggested analyses. We would like to stress that we use SDPs instead of individual SNPs and evaluate only two genotypes within our cross (equaling ±15 replicates at every assessed locus, with each line being inbred for decades to reach genome-wide homozygosity), providing sufficient power for local and distant QTL mapping. This comes at reduced QTL resolution (± 1600 SDPs vs ± 3M SNPs) - i.e., identified QTLs can span large chromosomal regions of sizes up to several Mb (average size of an SDP is 0.75 Mb). This makes it challenging to pinpoint the causal variant within a given locus, but the locus effect is very robust.

Within the HXB/BXH RI panel, the statistical power to detect QTLs with effect sizes above 0.25 (the median effect size in our study) is close to 1 (*Petretto et al., PLOS Genetics 2006*). Only for effect sizes lower than 0.11, which corresponds to the 5% of identified QTLs in our study, the power is 0.8 when the heritability is equal to 0.506 (here, heritability is defined as the ratio of genetic and environmental variance and in our panel these values are 0.506 and 1 respectively). Therefore, some cases will miss significance and could be considered as false negatives. We realize this is an important point for the potential interpretation of trait- or tissue specificity and have now better acknowledged this in the revised version of the manuscript (p7-8, p38 and p60).

2. For cases of discrepancies between QTL types (specifically, tissue specificity and ribo-QTLs that are not eQTLs), what fraction of QTLs show an effect in the same direction in the two data types? If, as a group, these QTLs really are specific, the direction of effect in the data in which no QTL was detected is expected to be random with respect to the direction of the detected QTL. By contrast, an excess of shared direction would point to more sharing of signal than can be detected using stringent QTL detection thresholds. These analyses should be added to the paper.

Following the Reviewer’s suggestion, we have addressed this point in the manuscript and added a new figure (Supplementary Figure 2B, p60) to cover the differences in direction and strength of effect size for: a) tissue-specific teQTLs; and b) category-specific expression and ribosome occupancy QTLs in heart. In each figure, blue and red bars point to positive and negative effect size values respectively. For tissue-specific QTLs (see figure below), whereas high resemblance in the direction of effect sizes is seen for local (*cis*) QTLs (82% of cases share direction), this appears completely random for the much more specific distant teQTLs (54%). Moreover, these tissue-specific QTLs exhibited a stronger effect size in the significant tissue, while the distribution of effect sizes remained constant for shared QTLs across both tissues.

**Distribution of effect sizes in tissue-specific and shared QTLs in heart and liver.** Bar plots with effect sizes for detected local (left) and distant (right) QTLs by tissue, sorted by effect size. Only three teQTLs were detected in liver and we therefore decided to only display heart-specific teQTLs in the right plot.

Regarding the genes with local QTLs detected as specific for mRNA expression (eQTL) or ribosomal occupancy (riboQTL), the corresponding figure (see below) illustrates that the sharing of direction in category-specific QTLs was indeed high (95%). This observation is in keeping with what we had tried to acknowledge in the manuscript (p7, lines 121-123) and had aimed to depict in Supplementary Figure 2A, p60. We are fully aware that some of the QTLs categorized as specific had a near-significance trend in the other expression layer and have now better indicated in the revised manuscript that some of these cases might be undetected false negatives due to limited power for low effect QTLs (p7-8, lines 134-141). We hope it is clear that, as we have illustrated, this does not lead to increased false-positive QTL detection, and most missed associations come with strongly reduced effect sizes, in particular for the tissue teQTL comparison between heart and liver.

**Distribution of effect sizes in local category-specific expression and ribosome occupancy QTLs and shared ones in the heart.** Bar plots with effect sizes for detected QTLs by category (eQTL, riboQTL or both), sorted by effect size.

Minor:

Page 18 line 377: I suggest removing the word "Unfortunately".

We have removed the word "Unfortunately" from this sentence.

**Reviewer #3:** In this manuscript, Witte et al. performed genotyping, RNA-seq and Ribo-seq in 30 rat recombinant inbred lines. Using this framework, they identify several eQTLs, ribo-QTLs and translational-efficiency QTLs (teQTLs). They detect a region in chromosome 3 that seems to be associated with the transcriptional efficiency of multiple genes in trans. They then confirm that finding using an engineered rat line carrying only the mutant segment compared to an isogenic control and proceed with some further characterization to show that the identified genetic variation in chromosome 3 induces a ribosomopathy leading to polysome half-mers. There are a few methodological concerns that need to be addressed:

1. The authors need to provide more details on how the recombinant inbred rat lines were generated and selected. What is the degree of relatedness between the different lines and how could that affect or confound results in QTL calling?

The HXB/BXH is a very well characterized and frequently used recombinant inbred panel that was established in the 80s by our collaborator and co-author Prof. Dr. Michal Pravenec (*Pravenec et al., J Hypertens 1989; Prinz et al., J Appl Physiol 2003*) and subsequently maintained within his laboratory at the CAS in Prague. The HXB/BXH RI strains have proven to be a valuable genetic tool for the identification of genotypic differences underlying physiological (cardiovascular and metabolic) and molecular traits. Many of these genetic studies have been carried out in, or in close collaboration with, our lab at the MDC Berlin (e.g., *Hubner et al., Nature Genet 2005; Pravenec et al., Nature Genet 2008; Petretto et al., Nature Genet 2008; Heinig et al., Nature 2010; McDermott-Roe et al., Nature 2011; Low & van Heesch et al., Cell Rep 2013; Rintisch & Heinig et al., Genome Res 2014*).

We and others have previously investigated the genetic architecture and level of relatedness across the RI lines (e.g., *Jirout et al., Mamm Genome 2003; Printz et al., J Appl Physiol 2003; STAR Consortium Nat Genet 2008; Simonis et al., Genome Biol 2012*). As suggested by the Reviewer, we have repeated these efforts using our latest (much higher resolution) genotype map that was generated specifically for this paper, by assessing the covariance of the genotypes across all 30 lines (see heatmap below, also added to Supplementary Figure 1, p58), as well as the frequencies of levels of relatedness (see histogram below, also added to Supplementary Figure 1, p58). The average covariance and distribution were as expected (0.506), although two pairs of genotypes display increased levels of relatedness: HXB17/HXB18 (0.736) and HXB3/HXB15 (0.804).

**Covariance of genotypes across all 30 RI lines.** Heatmap (left) and histogram (right) with correlations of covariances of the genotypes across all 30 recombinant lines. The average level of recombinant inbred relatedness is 0.506.

Taking relatedness into account and following the reviewer's suggestion, we evaluated the robustness of our eQTLs by comparing them with the results obtained by repeating the MatrixEQTL (*Shabalin et al., Bioinformatics 2012*) run for left ventricle including genotype relatedness information. We computed the eigenvectors of the covariance matrix of the genotypes and ran MatrixEQTL with 10 covariates set to the first 10 eigenvectors of the genotype covariance matrix. Next, we compared the effect sizes of both QTL datasets (with and without genotype covariance matrix information). The QTLs and effect sizes are almost identical (see scatter plots below) and the described distant translational master regulator located at chromosome 3 remained highly significant (p-value = 3.64 x 10^-8^). Methodological information on accounting for strain relatedness, the covariance results, as well as the scatter plots depicted below, were added to the revised manuscript as Supplementary Figure 1I+J+K+L, p58.

**Correlation of effect sizes from originally calculated QTLs and QTLs corrected by genotype covariance.** Correlation scatter plots for heart QTL effect sizes calculated by standard QTL mapping (y-axis) and after correcting for covariate variables (x-axis). The high correlation indicates that these covariates did not significantly affect our data and were not included in further analysis.

2. Similar to the above, it is unclear whether the authors accounted for population stratification and relatedness in their QTL analyses. The authors should present analyses attempting to correct for those common confounders.

We kindly refer the Reviewer to the correction for relatedness and population structure at point 1 above. We hope it is clear from the above-cited literature that the HXB/BXH recombinant inbred panel was not designed to reflect the general population, but instead established as a genetic tool from which trait variability can be used to find mechanistic links specific to this disease model. As this panel consists of only two genotypes it is difficult to extend our findings (e.g., gene-specific modes of regulation) to those found in outbred (rat) populations.

3. Also accounting for cryptic confounding using either PEER or SVA applied to the molecular data would be advised in such datasets. The number of identified QTLs seem out of proportion to the number of test subjects which could be partially accounted for by the decrease in multiple testing burden by aggregating variants in SDAs but could also suggest inflation from uncontrolled confounding.

We thank the Reviewer for pointing out the cryptic confounding issue for QTL detection. The Reviewer correctly acknowledges that various factors specific to our system (inbred lines, only two parental genotypes, genotypes collapsed into SDPs) might increase our power to detect QTLs and thereby the number of QTLs we detect and report.

As suggested, we have evaluated the effect of cryptic confounders within our data by running the R package SVA (*Leek et al., Bioinformatics 2012*). For each category (eQTL, riboQTL, teQTL), we selected the optimal number of significant hidden surrogate variables (5 to 7 depending on the analysis) and ran MatrixEQTL using these surrogates as covariates. As done before to assess the effect of relatedness on QTL mapping, we compared the effect sizes of both QTL mappings (with and without SVA surrogate matrix information). Again, the correlations based on the effect sizes of local QTLs are significant (Pearson’s r = 0.99, see scatter plots below), indicating that none of the predicted confounding factors affected the effect sizes and the significance of the reported QTLs. This figure was also added to Supplementary Figure 1, p58, in the revised manuscript.

**Correlation of effect sizes from originally calculated QTLs and QTLs corrected by hidden confounders.** Correlation scatter plots for heart QTL effect sizes calculated by standard QTL mapping (y-axis) and after correcting for surrogate variables (x-axis). Distant QTLs were not included as this correction can negatively impact the detection of *trans* loci that associate with multiple genes. The high correlation indicates that these surrogate variables did not significantly affect our data and were not included in further analysis.

Based on what has been described previously (*Fusi et al., PLOS Comp Biol 2012; Stegle et al., PLOS Comp Biol 2010; Stegle et al., Nat Protoc 2012*), we decided against accounting for hidden confounders for the identification of distant QTLs. These and other studies showed that correcting for hidden variables of confounders has a negative impact on the detection of *trans* loci that associate with multiple genes. This especially concerns distant QTL hotspots, which we were primarily interested in detecting and characterizing during this study. When correcting for confounding variability, hotspots can be explained away as a global factor, which is ill-advised for *trans* QTL studies. Instead, we applied two independent methods (Matrix EQTL and HESS, the latter was specifically devised for the detection of weak *trans* effects) for increased sensitivity and independent validation of distant QTL hotspots. Moreover, for the Chromosome 3 teQTL - the main focus of our work - we made use of two separate congenic lines in which this particular locus was fully isolated and used independent technologies (Ribo-seq and polysome profiling) to evaluate the translational phenotype. This was a significant effort and these lines fully and independently recapitulate our distant QTL mapping results.

4. In addition, read mapping errors due to cross-mappability could explain some/all of the detected trans-QTLs. The authors should check for cross-mappability of their trans-eGenes with any cis-genes and present read-pileups for cross-mappable genes stratified by genotype of the corresponding trans SDA to reassure that is not the case. [PMID: 30613398]

As suggested by the reviewer, we have checked for the potential of cross-mapping in the set of identified *trans* QTLs in heart and liver. The method included in the shared reference was intended to analyze exon and UTR sequences (*Saha and Battle, F1000Res 2018*), hence we adapted it by developing an ‘in-house’ algorithm that can extract CDS k-mers (length=29bp) and identify if any of these k-mers map to exon sequences (CDS or UTR; number of mismatches = 0-2) from different genes so as to define cross-mappable pair of genes in rat. We found that cross-mappability had a rather negligible effect on our analysis as only 1 out of 160 distant QTLs (including eQTLs, riboQTsL and teQTLs) were cross-mappable with a *cis*-gene in the same SDP: the eQTL for a gene named *AABR07002683.1*. Reassuringly, none of the genes with distant teQTLs were affected, meaning that our work on the teQTL hotspots - including the chromosome 3 teQTL - was not influenced by cross-mappability. We thank the Reviewer for this useful suggestion and have now removed the aforementioned cross-mappable heart eQTL for *AABR07002683.1* from our results and tables. We added this analysis and findings to the Methods section (p46, lines 849-856).

5. Do the QTLs identified in this dataset replicate in other rat QTL datasets? That would provide some more confidence in the findings

The classical eQTLs studied previously within the same tissues of the same HXB/BXH panel (e.g., *Cyp17a1* and *Serpina3m* (*Low et al., Cell Rep 2013*) or *Endog* (*McDermott-Roe, Nature 2011*)) replicate well within our data. However, there is no other rat system that has mapped translation QTLs to date. Because we realize replication of teQTLs is crucial in establishing confidence of QTL identification, we independently validated our main QTL results within two congenic rat lines, with two independent techniques (ribosome profiling and polysome profiling). These lines, which differ only in genotype for our QTL region of interest, fully replicate our teQTL results in each of the performed experiments. Importantly, these comparisons reflect direct strain vs. strain comparisons. The generation of the congenic lines took repeated backcrosses for nearly 10 generations to isolate this specific segment on a different background.

To investigate the replicability of our translation QTLs a bit further, we also re-evaluated the effect of biological variability within the replicated liver translatome datasets of two specific RI strains (BXH12 and BXH13). Next to the excellent biological replicability across these triplicates (Pearson’s r ~ 1, Figure S1C, p58), genes that were defined as riboQTLs within loci with divergent genotypes among both strains showed a much lower adjusted p-value than the rest of genes in a direct comparison (0.011 vs 0.56, see figure below). However, we acknowledge that these replication analyses do not ensure full QTL mapping reproducibility in a completely independent rat model system (only for a subset of loci and for a selection of RI lines). Hence, through the use of stringent adjusted p-values for QTL detection, we tried to minimize the chance of false-positive detection.

**Biological replicability across triplicates.** Density plot illustrating the differences in significance of differential gene expression between BXH12 and BHX13, both of them were biologically replicated three times. For genes defined as riboQTLs and with divergent genotype between BXH12 and BXH13 (blue), the p-values are much lower than for the rest of genes (red). For this analysis, only *cis* effects are taken into account.

6. Allowing multimapping regions in detecting snoRNAs is ill-advised as it further raises concern for the accuracy of QTL detection for those transcripts.

We apologize for this misunderstanding regarding the read mapping and quantification methods used in our manuscript. Any data quantification that included multimapping reads were only performed post-QTL mapping, with the single goal to investigate the genome-wide expression of all snoRNAs, to make sure high sequence identity between duplicated snoRNAs did not make us blind to highly expressed or differentially regulated snoRNAs in addition to *SNORA48*. We observed that 406 snoRNAs have duplicated homologs that share 100% of sequence identity and hence all reads would map ambiguously and would be automatically omitted from gene quantification. This includes *SNORD24*, which is located in the intron of *Rpl7a* in a highly homologous segmental duplication within the chr3 teQTL locus, thus rendering this snoRNA as 'non-expressed'. *SNORD24* was previously associated with a molecular phenotype highly similar to the one described in our paper (but then in yeast), and by allowing multimapping in a subsequent quantification of all snoRNAs genome-wide, we wanted to make sure we captured all potential differentially regulated snoRNAs associated with our QTL, including *SNORD24*. To be clear - none of the presented QTL mapping analyses were performed on non-uniquely mapping reads. We have now repeated the snoRNA expression quantification requiring reads to map uniquely -as we did for QTL detection- and have masked known identical snoRNA duplications. The end result is highly similar to the presentation of these data in our paper:

**Effect of multiple mapped reads in the quantification of snoRNA expression and significance of SNORA48.** Scatter plots showing expression levels of all cardiac-expressed snoRNAs as measured by totRNA-seq and Ribo-seq data, with *SNORA48* highlighted in pink. For both Ribo-seq datasets, *p*-value volcano plots show the significance of the differential regulation of *SNORA48* (highlighted in pink). Left panel includes multiple mapped reads and right panel only includes uniquely mapped reads. The use of multiple mapped reads had a negligible effect in the differential expression of snoRNAs, but only uniquely mapped reads were considered in the revised manuscript.

Furthermore, we have changed this figure in the revised version of our manuscript and have clarified the manuscript methods section on read mapping, quantification and QTL mapping.

7. This is especially relevant for SNORA48 that the authors propose as the mechanism for the observed ribosomopathy in the chromosome 3 locus. The case for it being the cause of the ribosomopathy seems weak with the only supporting data being its decreased ribosome association in mutant hearts. Does overexpression of the target RNA in SHR-BN-(3S) rats rescue the observed ribosomopathy?

The association with *SNORA48* was obtained through QTL mapping analyses for which only uniquely mapping reads were considered. The use of multi-mapped or uniquely mapped reads for posterior snoRNA quantification did not have any effect on the significance of the differential expression of *SNORA48*, but just helped to show that *SNORA48* is, independently of the mapping approach, the most highly expressed snoRNA in rat hearts and the only one that is differentially expressed when compared to the rest of snoRNAs (please see the figure at comment 6). We are hypothesizing that the misregulation of *SNORA48* is a key component in the chain of events that drives the ribosomopathy, as it is the only regulatory RNA that associates with the QTL, it is the most highly expressed snoRNA in rat hearts (hence likely important), and snoRNAs are important regulators of ribosomal RNA processing. Furthermore, the QTL solely becomes apparent in the Ribo-seq, and not the RNA-seq data. In agreement with the comment made by Reviewer 2 above, we completely agree that these findings will prompt additional research into this small RNA. Ideally, a possible validation experiment would involve the *in vivo* rescue of the translational efficiency phenotype through the overexpression of a snoRNA in rat hearts. Although worthy of follow-up investigations in future studies, we believe this is beyond the scope of the current manuscript.

8. Minor comment: Co-localization has evolved to have a specific meaning implying a shared causal signal between two different datasets. The authors use it to mean positional overlap. Would suggest rephrasing throughout.

We thank the Reviewer for this correction and have changed this throughout the manuscript.

**Second round of review**

**Reviewer 2**

Thanks to the authors for addressing my comments (I was Reviewer #2 in the previous submission). My remaining comments are on the authors’ description of heritability and power.

1. “Here, heritability is defined as the ratio of genetic and environmental variance” (line 924). This is incorrect: heritability is the ratio of genetic and total (not environmental) variance. This statement needs to be corrected, along with any calculations that may be based on this incorrect description.

2. It is not clear what units the effect size in this section (i.e., 0.25 in line 926) is expressed in. Standard deviations? Fraction of total variance? TPMs? Read counts? This is important for interpreting the statistical power of this dataset.

3. How were the genetic and environmental variances calculated?

4. Different genes have different heritabilities, so what does the value of 0.506 (line 924) mean? Is this an average? Median? Something else?

Finally, I do want to compliment the authors for their patient and level-headed response to Reviewer #1, whose unnecessarily rude review betrayed profound confusion about both this paper specifically and modern genetics and genome biology in general.

**Reviewer 3**

The authors did a nice job in this revision to address the reviewers’ comments and criticisms by undertaking additional computational analyses. There are still a few questions that remain:

1. The authors provided more details on the population structure of their 30 inbred rat lines. Not surprisingly, there is a substantially high degree of relatedness between the different lines used in their QTL calling. They show that accounting for up to 10 PCs doesn’t change their QTL findings. It is questionable whether a PCA approach would appropriately account for relatedness in this small sample size and highly related population (see Price et al. Nat Rev Genetics 2010;11:459-463 and a recent simulation study by Yao et al. bioRxiv 2019;858399). The authors need to use a mixed model approach to account for kinship and population structure instead. If that is not computationally tractable for all QTL analyses (mixed models are known to be rather slow to fit), it should at least be done as a sensitivity analyses for significant QTLs to provide peace of mind that the results are not affected by relatedness.

2. The authors used SVA to estimate hidden confounders and effectively showed that SVA correction did not significantly influence their cis-QTL results. The authors then went on to claim that they decided to avoid that approach in their trans-QTL data since inference of surrogate variables can lead to elimination of true trans-QTL hotspots. Although that is true, since trans-QTL hotspot detection is highly susceptible to batch effects, the genomics community, including most large consortia like GTEx have opted for a conservative approach of including hidden confounders in their trans-QTL analyses to avoid any potential false positives (see Nature 2017;550:204-213 for a detailed discussion of the matter). Therefore, the reviewer believes that the results with PEER/SVA correction for trans-QTLs should still be presented as sensitivity analysis in the manuscript. Even if the approach might eliminate some true hotspots, presenting and discussing which trans-QTLs are robust to confounder evaluation would be very informative for future studies looking into those results for downstream experimentation.

**Reviewer 4**

I would like to start by acknowledging the fact that I do not have the proper expertise on OTL mapping, but I trust that reviewers 2 and 3 can address the data relevant to these sections. I can only comment on the ribosome profiling data.

While reviewer 1 raised some valid concerns regarding the authors’ interpretation of the ribosome profiling, in my opinion this does not detract from the impact and the contributions of the manuscript on/to the translation field. In particular, I found the effects of the CDS-length on TEs to be very convincing and appears to be evolutionary conserved. Also of note is the authors analysis of previous published data to come up with a “unified” model for how defects in the translation machinery can lead to decrease in translation for long CDSs.

Having said that, I suggest that the authors tune down their interpretation of the profiles in Figure 3. I am not convinced that the authors can identify the source of the differences in translation induced by the 3p teQTL by mere examination of profiles in Figures 3A, B, C (I believe this what reviewer 1’s main concerns were). The accumulation of “half-mer” polysomes appears to be too low to be significant. The authors claim that based on the half-mer formation that there must be defects in translation initiation; however this cannot explain why the levels of the 40S and 60S subunits remain unchanged for the teQTL SHR relative to the BN one. Instead, I would argue that the most convincing observation from the profile is the accumulation of higher-order polysomes, which can be interpreted as defects in termination or recycling; alternatively this can be interpreted by increased translation rates (perhaps for short CDS mRNAs). At the end, I agree with the authors there must be some differences in translation between the two QTLs, but the current data cannot address whether this is due to differences in translation initiation or other phases of translation.

**Authors Response**

**Point-by-point responses to the reviewers’ comments:**

**Reviewer #2:** The online reviewer system gives me two boxes for author comments, so I am reproducing my comments here. Apologies if they show up twice.

Thanks to the authors for addressing my comments (I was Reviewer #2 in the previous submission). My remaining comments are on the authors' description of heritability and power.

1. "Here, heritability is defined as the ratio of genetic and environmental variance" (line 924). This is incorrect: heritability is the ratio of genetic and total (not environmental) variance. This statement needs to be corrected, along with any calculations that may be based on this incorrect description.

We thank the Reviewer for this correction. We have corrected this statement in the manuscript and we have updated the calculation of heritability, standardized effect size and power (Section ‘Detection of tissue-specific and recurrent QTLs’: lines 970-989). We realize that this section was not sufficiently explained and we have provided new details on how trait heritability was calculated (Section ‘Detection of tissue-specific and recurrent QTLs’: lines 970-975). After updating the calculations, the statistical power to detect QTLs (including tissue-specific QTLs) with standardized effect sizes above 0.7 - the median value in our study, see point 2 below for more information - is equal to 1. The estimated power remained close to 1 for lower standardized effect sizes than the median, and it was ~0.7 for standardized effect sizes lower than 0.55 (5^th^ percentile of the distribution of QTL effect sizes).

2. It is not clear what units the effect size in this section (i.e., 0.25 in line 926) is expressed in. Standard deviations? Fraction of total variance? TPMs? Read counts? This is important for interpreting the statistical power of this dataset.

The (standardized) effect sizes are fractions that represent the difference in average expression (mRNA abundance, ribosome occupancy or TE) values per gene between homozygotes, as a proportion of the total genetic variance. These values, along with calculated heritabilities, can be included in a tool specifically designed to calculate power for RI lines (http://power.genenetwork.org/). To calculate these standardized effect sizes we have used a linear mixed model (‘lme4qtl’) that can account for relatedness and population structure (as suggested by Reviewer 3). For each trait, we used the R function ‘VarProp’ on the built linear mixed models, which estimates the fraction of variance explained by the genetic difference between homozygotes, and we used these generated values as standardized QTL effect sizes for the power calculations.

3. How were the genetic and environmental variances calculated?

Narrow-sense heritabilities were calculated using the simplified formula in Petretto et al. (*PLoS Genet*. 2006; 20;2(10):e172), based on the method of Hegmann and Possidente (*Behav Genet.* 1981;11:103–114): h^2^_trait_ = 0.5V_A_/(0.5V_A_ + V_E_), where V_A_ is the additive genetic component and represents the variance of the strain means (i.e., between biological replicates), and V_E_ is the environmental component and represents the average variance across all strains. In the example below we show the coefficient of variations (standardized dispersions) within and between Ribo-seq expression levels of two of the RI strains (BXH12 and BXH13, 3 replicates each; *see also Figure S1C*), as well as for the Ribo-seq expression levels of the whole RI panel in liver and left ventricle. The coefficient of variation within each strain was lower than the coefficient of variation of the two strains and the coefficient of variation of the 30 RI strains in the two considered tissues (a similar trend was observed when looking at intra- *vs* inter-strain variances):

**Within- and inter-strain coefficients of variation.** Violin plots with coefficients of variation of ribosome occupancy values for BXH12 (3 replicates), BXH13 (3 replicates), BXH12 and BXH13 (BXH12+BHX13, 3+3 replicates), and all 30 RI strains (liver -liv- and left ventricle -lv-). Values were calculated using the ‘cv’ R function (package ‘goeveg’).

4. Different genes have different heritabilities, so what does the value of 0.506 (line 924) mean? Is this an average? Median? Something else?

We initially reported a single value representing the average heritability across all QTLs. In the revised manuscript we have included a figure (Additional File 1: Figure S2C, see below) displaying the range of heritabilities for all genes and genes with QTLs.

**Estimated narrow-sense trait heritabilities.** Histograms with trait heritability values estimated for all expressed genes and QTL genes.

Finally, I do want to compliment the authors for their patient and level-headed response to Reviewer #1, whose unnecessarily rude review betrayed profound confusion about both this paper specifically and modern genetics and genome biology in general.

We would like to thank the Reviewer for her/his support during the Reviewing process and helpful comments that greatly improved the presentation of the results.

**Reviewer #3:** The authors did a nice job in this revision to address the reviewers' comments and criticisms by undertaking additional computational analyses. There are still a few questions that remain:

We are very grateful to the Reviewer for these positive comments.

1.	The authors provided more details on the population structure of their 30 inbred rat lines. Not surprisingly, there is a substantially high degree of relatedness between the different lines used in their QTL calling. They show that accounting for up to 10 PCs doesn't change their QTL findings. It is questionable whether a PCA approach would appropriately account for relatedness in this small sample size and highly related population (see Price et al. Nat Rev Genetics 2010;11:459-463 and a recent simulation study by Yao et al. bioRxiv 2019;858399). The authors need to use a mixed model approach to account for kinship and population structure instead. If that is not computationally tractable for all QTL analyses (mixed models are known to be rather slow to fit), it should at least be done as a sensitivity analyses for significant QTLs to provide peace of mind that the results are not affected by relatedness.

We thank the Reviewer for pointing out the population structure issue for the robust detection of QTLs. In this updated version of the manuscript we have built a linear mixed model to jointly account for kinship relatedness and population structure. We have included the details of the approach in the section ‘Pairwise association testing using Matrix eQTL’ in the Methods (lines 946-959). Population structure has been inferred by running fastSTRUCTURE (Raj et al. Genetics. 2014;197(2):573–589), stratifying the set of 30 RI individuals in 5 different subpopulations, and we have used the generated allele frequencies to calculate global and subpopulation heterozygosities and subsequently estimate allele fixation indices, including these parameters along with the calculated kinship matrix in the ‘lme4qtl*’* mixed model. For 75% of *cis* and 83% of *trans* QTLs, the significance of the quantitative effect calculated by matrix eQTL is also reported by the linear mixed model, reassuring the robustness of our approach to identify QTLs. Importantly, all the genes whose translation rates were controlled by the *trans* QTL hotspot in chromosome 3 were significant after correcting for relatedness. We have incorporated the evidence of statistical significance for both approaches in the Supplementary Material (Table S2).

2.	The authors used SVA to estimate hidden confounders and effectively showed that SVA correction did not significantly influence their cis-QTL results. The authors then went on to claim that they decided to avoid that approach in their trans-QTL data since inference of surrogate variables can lead to elimination of true trans-QTL hotspots. Although that is true, since trans-QTL hotspot detection is highly susceptible to batch effects, the genomics community, including most large consortia like GTEx have opted for a conservative approach of including hidden confounders in their trans-QTL analyses to avoid any potential false positives (see Nature 2017;550:204-213 for a detailed discussion of the matter). Therefore, the reviewer believes that the results with PEER/SVA correction for trans-QTLs should still be presented as sensitivity analysis in the manuscript. Even if the approach might eliminate some true hotspots, presenting and discussing which trans-QTLs are robust to confounder evaluation would be very informative for future studies looking into those results for downstream experimentation.

Following the Reviewer’s suggestion, we have corrected *trans*-QTLs for hidden confounders in our revised manuscript using both SVA and PEER. As expected, the correlation of absolute effect sizes before and after correcting for confounding covariates is lower for distant riboQTLs and teQTLs associated with *trans*-acting QTL hotspots, indicating that the detection of distant QTLs orchestrated by a single locus is more susceptible to the correction of hidden confounders (see the figure below; *new Figure S1K*). While correcting for hidden covariates may be strongly necessary for some studies, especially for data coming from human populations, the generation of the RI panel allows for the control of many of the confounders -for example, rats are kept in a controlled environment and processed for sequencing on the same day-. Of note, 76.47% of the distant teQTLs are significant (albeit to a lower extent) after correcting for relatedness and hidden covariates. This includes genes associated with 8 out of 9 teQTL hotspots, importantly all the genes associated with the chromosome 3p teQTL locus.

We have included the evidence of statistical significance after correction for hidden covariates in the Supplementary Material (Table S2) and have added discussion about the potential implications of correcting for hidden variables that may 'explain away' distant hotspots. Although most of our teQTLs are still detected after correction, we do believe that such corrections can contribute to the unwanted camouflaging of potentially disease-relevant genetic associations.

**Correlation of effect sizes from originally calculated QTLs and QTLs corrected by hidden confounders.** Correlation scatter plots for heart QTL effect sizes calculated by standard QTL mapping (y-axis) and after correcting for surrogate variables (x-axis).

**Reviewer #4:** I would like to start by acknowledging the fact that I do not have the proper expertise on OTL mapping, but I trust that reviewers 2 and 3 can address the data relevant to these sections. I can only comment on the ribosome profiling data.

While reviewer 1 raised some valid concerns regarding the authors’ interpretation of the ribosome profiling, in my opinion this does not detract from the impact and the contributions of the manuscript on/to the translation field. In particular, I found the effects of the CDS-length on TEs to be very convincing and appears to be evolutionary conserved. Also of note is the authors analysis of previous published data to come up with a “unified” model for how defects in the translation machinery can lead to decrease in translation for long CDSs.

Having said that, I suggest that the authors tune down their interpretation of the profiles in Figure 3. I am not convinced that the authors can identify the source of the differences in translation induced by the 3p teQTL by mere examination of profiles in Figures 3A, B, C (I believe this what reviewer 1’s main concerns were). The accumulation of “half-mer” polysomes appears to be too low to be significant. The authors claim that based on the half-mer formation that there must be defects in translation initiation; however this cannot explain why the levels of the 40S and 60S subunits remain unchanged for the teQTL SHR relative to the BN one. Instead, I would argue that the most convincing observation from the profile is the accumulation of higher-order polysomes, which can be interpreted as defects in termination or recycling; alternatively this can be interpreted by increased translation rates (perhaps for short CDS mRNAs). At the end, I agree with the authors there must be some differences in translation between the two QTLs, but the current data cannot address whether this is due to differences in translation initiation or other phases of translation.

We thank the new Reviewer for being willing to evaluate our work and for the constructive feedback. We tuned down our conclusions on the accumulation of half-mer polysomes as the prime cause of the translational defect and adapted a more general description of our observations that the polysome profiles display differences in ribosome association between both rat strains. We now specifically mention the accumulation of higher order polysomes and discuss the possibility that a different defect during phases other than translation initiation may be at the basis of the Chr3 teQTL.

**Third round of review**

**Reviewer 2**

No more comments. Thanks to the authors for a job well done.
